# Wnt signal-dependent antero-posterior specification of early-stage CNS primordia modeled in EpiSC-derived neural stem cells

**DOI:** 10.3389/fcell.2023.1260528

**Published:** 2024-02-09

**Authors:** Kae Nakamura, Yusaku Watanabe, Claire Boitet, Sayaka Satake, Hideaki Iida, Koya Yoshihi, Yasuo Ishii, Kagayaki Kato, Hisato Kondoh

**Affiliations:** ^1^ Faculty of Life Sciences, Kyoto Sangyo University, Kita-ku, Kyoto, Japan; ^2^ Université Joseph Fourier, Domaine Universitaire, Saint-Martin-d’Hères, France; ^3^ Department of Biology, School of Medicine, Tokyo Women’s Medical University, Tokyo, Japan; ^4^ National Institute for Basic Biology, Okazaki, Aichi, Japan; ^5^ Biohistory Research Hall, Takatsuki, Osaka, Japan

**Keywords:** epiblst, CNS primordia, anteroposterior regionalization, CNS subdivisions, Wnt signaling

## Abstract

The specification of the embryonic central nervous system (CNS) into future brain (forebrain, midbrain, or hindbrain) and spinal cord (SC) regions is a critical step of CNS development. A previous chicken embryo study indicated that anterior epiblast cells marked by *Sox2* N2 enhancer activity are specified to the respective brain regions during the transition phase of the epiblast to the neural plate-forming neural primordium. The present study showed that the SC precursors positioned posterior to the hindbrain precursors in the anterior epiblast migrated posteriorly in contrast to the anterior migration of brain precursors. The anteroposterior specification of the CNS precursors occurs at an analogous time (∼E7.5) in mouse embryos, in which an anterior-to-posterior incremental gradient of Wnt signal strength was observed. To examine the possible Wnt signal contribution to the anteroposterior CNS primordium specification, we utilized mouse epiblast stem cell (EpiSC)-derived neurogenesis in culture. EpiSCs maintained in an activin- and FGF2-containing medium start neural development after the removal of activin, following a day in a transitory state. We placed activin-free EpiSCs in EGF- and FGF2-containing medium to arrest neural development and expand the cells into neural stem cells (NSCs). Simultaneously, a Wnt antagonist or agonist was added to the culture, with the anticipation that different levels of Wnt signals would act on the transitory cells to specify CNS regionality; then, the Wnt-treated cells were expanded as NSCs. Gene expression profiles of six NSC lines were analyzed using microarrays and single-cell RNA-seq. The NSC lines demonstrated anteroposterior regional specification in response to increasing Wnt signal input levels: forebrain-midbrain-, hindbrain-, cervical SC-, and thoracic SC-like lines. The regional coverage of these NSC lines had a range; for instance, the XN1 line expressed Otx2 and En2, indicating midbrain characteristics, but additionally expressed the SC-characteristic Hoxa5. The ranges in the anteroposterior specification of neural primordia may be narrowed as neural development proceeds. The thoracic SC is presumably the posterior limit of the contribution by anterior epiblast-derived neural progenitors, as the characteristics of more posterior SC regions were not displayed.

## Introduction

CNS development initiates when the Sox2 N2 enhancer is activated in the anterior epiblast (Iwafuchi-Doi et al., 2011). The N2 enhancer was identified in a systematic survey of an array of chicken enhancers regulating the *Sox2* gene expression and characterized as the one that is activated first among the enhancers and in the entire anterior epiblast preceding the neurogenesis-associated *Sox2* expression (Uchikawa et al., 2003). The N2 enhancer sequence is located upstream of the *Sox2* gene (∼4 kb in the mouse, ∼3 kb in the chicken, and ∼1.5 kb in zebrafish) (Uchikawa et al., 2004; Kamachi et al., 2009; Okamoto et al., 2015), and is conserved from mammals to fish in the regulation and function during the developmental stages from anterior epiblast to the early neural tube development. The N2 enhancer sequence is included in the telencephalic enhancer of later embryonic stages determined by Zappone et al. (2000), suggesting that N2 activity becomes later involved in the regulation of telencephalic development.

The N2 enhancer is activated by the combined action of Otx2, Zic2/3, and Pou3/5 transcription factors (TFs) (Iwafuchi-Doi et al., 2012). Because the Zic2/3 (Elms et al., 2004; Iida et al., 2020) and Pou3/5 genes (Lavial et al., 2007; Iwafuchi-Doi et al., 2012) are more widely expressed than Otx2 (Chapman et al., 2002; Iwafuchi-Doi et al., 2012) in both mouse and chicken embryos, the activity distribution of the N2 enhancer reflects Otx2 expression. The mouse N2 enhancer sequence was regulated correctly in the chicken and zebrafish embryos concerning the spatial distribution of the enhancer activity in the anterior epiblast, neural plate, and nascent neural tubes (Iwafuchi-Doi et al., 2011). The loss of the N2 enhancer activity in the mouse embryos (targeted deletion) and in zebrafish [*Pou5f2* (*Spg*) mutation] resulted in the loss of the *Sox2* activation in the anterior epiblast (Iwafuchi-doi et al., 2011).

In chicken embryos, the N2 enhancer is activated in the anterior epiblast from the start, at early st. 5 in chicken embryos (∼20 h of incubation) (Uchikawa et al., 2003; Yoshihi et al., 2022a). In contrast, in mouse embryos, the N2 is activated in the entire epiblast at E6.5, but after E7, the N2 enhancer activity is cleared in the posterior epiblast, presumably reflecting the loss of Otx2 expression in the region (Iwafuchi-Doi et al., 2012), indicating that N2 enhancer-dependent *Sox2* regulation becomes similar to the chicken embryo from E7.

A critical feature of the N2-dependent *Sox2* expression at the anterior epiblast stage is that the *Sox2* expression occurs in the medial region of the N2 enhancer-active epiblast that approximates the neural plate-forming region of the anterior epiblast, leaving more distal region corresponding roughly the head ectoderm-forming region of the anterior epiblast (Iwafuchi-Doi et al., 2011; Yoshihi et al., 2022b). However, the entire anterior epiblast with the N2 enhancer activation has the potential to develop into the neural plate, as the anterior mesendoderm was grafted at the periphery of the anterior epiblast elicited the *Sox2*-expressing secondary brain development from the initially *Sox2*-negative epiblast (Yoshihi et al., 2022a). This secondary brain development does not occur in the posterior epiblast lacking the N2 enhancer activation. Taken together, the N2 enhancer activation potentiates neural Sox2 expression.

A previous study using chicken embryos with randomly EGFP-labeled epiblast cells and tracing back the initial epiblast positions of the forebrain (FB), midbrain (MB), and hindbrain (HB) precursors (Yoshihi et al., 2022a) indicated that these precursors are arranged in anterior-to-posterior order in the ∼700 µm anteroposterior span in the N2 enhancer-active anterior epiblast. These brain portion precursors then converge toward the midline and form the neural plate at st. 6 (∼25 h of incubation), and CNS development starts (Yoshihi et al., 2022a). These brain portion precursors in the anterior epiblast were regionally specified at least grossly. This specification was indicated by the experiments in which a second convergence site for the brain precursors was created by transplanting anterior mesendoderm (AME) ectopically; it gathered only a fraction of the CNS-portion precursors. The second brain consisted only of the portions where the ectopic AME successfully gathered the precursors (Yoshihi et al., 2022a).

The situation of the mouse embryo seems similar. A cell tracing study in mouse epiblasts indicated that FB, MB, and HB precursors are arranged in anterior-to-posterior order (Cajal et al., 2012). A study using embryonic stem cell-derived neurogenesis *in vitro* indicated that regional specification of the CNS occurs at the epiblast stage (Metzis et al., 2018). Moreover, analysis of randomly labeled cell clones in mouse embryos showed the presence of clones encompassing the brain and anterior spinal cord, supporting the model of anterior epiblast-derived anterior spinal cord tissue (Tzouanacou et al., 2009). One difference is that the brain precursors do not converge onto the midline in mouse embryos (Cajal et al., 2012), presumably reflecting the smaller embryonic width (∼2 mm in chicken embryos vs. ∼200 µm in mouse embryos).

In these previous studies, the focus was on the development of brain portions. However, the present study demonstrates that the anterior spinal cord also originates from the N2-activated anterior epiblast region. The positioning of the spinal cord precursors in the anterior epiblast and their trajectories during the embryonic CNS are analyzed in the first part of the Results section. The anterior epiblast-derived spinal cord tissue occupies the anterior spinal cord. In contrast, the posterior part of the spinal cord develops from neuromesodermal progenitors (NMPs) originating from the posterior epiblast (Takemoto et al., 2006; Takemoto et al., 2011; Kondoh and Takemoto, 2012). NMPs are labeled by the activity of the N1 enhancer, identified in the same *Sox2* enhancer screening as N2 (Uchikawa et al., 2003), and shown to be activated by the combined action of Wnt and Fgf signaling (Takemoto et al., 2006; Takemoto et al., 2011). Additionally, NMPs at the stage of determining the neural or mesodermal fate of the posterior epiblast are characterized by the coexpression of Sox2 and Bra (T) (Kondoh et al., 2016). However, NMPs are beyond the scope of this study.

Given all this, how does the anterior-to-posterior regional specification of the CNS precursor occur in the anterior epiblast? In chicken embryos, Wnt8c and other Wnt ligands are expressed along the primitive streak in the posterior half of embryos (Chapman et al., 2002). In mouse embryos, Wnt3 is expressed in the posterior, amnion-proximal region at E7.5 (Lewis et al., 2008). We investigated the Wnt signal levels in individual cells of mouse embryos using the WntVis reporter, where the Wnt signal input levels are reflected by EGFP fluorescence intensity (Takemoto et al., 2016). In WntVis reporter embryos at E7.5, the anterior epiblast received Wnt signals in a graded fashion, with higher signal levels at more posterior positions (Takemoto et al., 2016), indicating that Wnt3 ligands emanating from the posterior epiblast created a gradient of Wnt signals received by the anterior epiblast cells. Thus, the differential Wnt signal input emerged as a candidate cue for the anteroposterior specification of the anterior epiblast. Metzis et al. (2018) also showed that culturing E7 mouse embryos in a high concentration (20 µM) of the Wnt agonist CHIR99021 (CHIR) caused anterior expansion of posterior SC-characteristic Cdx2 expression.

The next issue is capturing the initial stage of anteroposterior specification in the anterior epiblast or epiblast-derived earliest neural primordia. We took the approach of producing neural stem cell lines representing the epiblast-derived nascent neural primordium, taking advantage of the mouse epiblast stem cell (EpiSC) lines derived from the mouse epiblast (Brons et al., 2007; Tesar et al., 2007). EpiSC lines established from mouse epiblasts are maintained in culture media containing activin and FGF2. Activin is an experimental replacement for Nodal in mouse embryos that inhibits neural development (Camus et al., 2006). Accordingly, removing activin from the EpiSC culture elicits neural development of the entire EpiSC population (Iwafuchi-Doi et al., 2012). The strategy we used was to capture the earliest stage of neural development in the form of NSC lines and investigate the impact of changing the Wnt signal input.

Regarding the underlying rationale of NSC line production, the culture conditions used to establish and maintain the stem cell lines that represent a transient cell state in embryos are considered to arrest the developmental progression of cells at a particular developmental stage and expand the cells of that stage. Classic examples include mouse ESCs, human ESCs, and mouse EpiSC lines representing the blastocyst inner cell mass, early postimplantation epiblast, and mature epiblast, respectively, which show the arrest of developmental progression of cells at a stage close to the culture-inoculated embryonic tissues (Evans and Kaufman, 1981; Brons et al., 2007; Tesar et al., 2007; Vallier et al., 2009; Matsuda et al., 2017). Neural stem cell (NSC) lines can be established from the embryonic CNS and adult CNS in culture media containing EGF and FGF2 (Tropepe et al., 1999; Ying et al., 2003; Louis et al., 2013). In addition, these NSC lines maintain the features of the original developmental stages and CNS regions, namely, those of the embryonic or adult stages (Louis et al., 2013) and the specific regions of the adult brain (Kallur et al., 2006). Our strategy was to apply neural stem cell-generating culture conditions to the nascent stage of neural primordia with the aim of establishing NSC lines carrying the characteristics of early CNS precursors, including anteroposterior regional specification.

In mouse embryos, the embryonic CNS is finely regionalized, as indicated by the specific expression patterns of TF genes by approximately E10 (Bulfone et al., 1993; Shimamura et al., 1995; Shimamura et al., 1997; Gray et al., 2004; Thompson et al., 2014; Berg et al., 2019; La Manno et al., 2021), and the embryonic CNS regions develop into the functional units of the adult CNS (Thompson et al., 2014; Zeisel et al., 2018; Berg et al., 2019). The initial anteroposterior regional specification precedes the later complex regional specification of the embryonic CNS. The approach we have taken will also be useful in deciphering the processes in the entire regional specification of the embryonic CNS.

## Results

### Distribution and migration of anterior epiblast-derived anterior spinal cord precursors in chicken embryos

**FIGURE 1:**
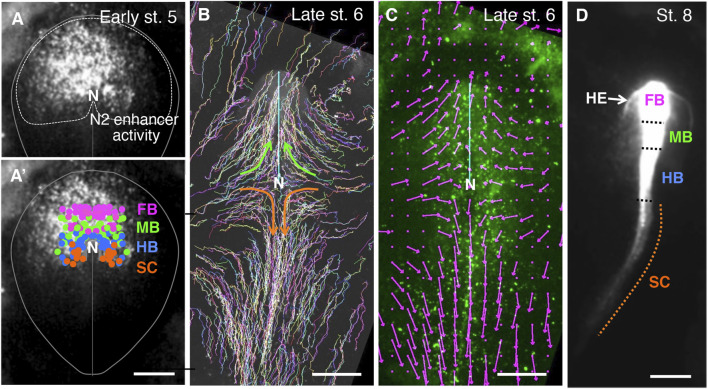
The early-stage setting of anteroposterior regionality in the CNS primordia, as indicated by the cell tracking in chicken embryos. **(A)** the *Sox2* N2 enhancer activity at st. 5 in an embryo electroporated with the N2-tkmCherry vector at st. 4 (Uchikawa et al., 2017). Because the mCherry fluorescence was recorded 2.5 h after vector electroporation, when the fluorescence became first detectable, the fluorescence appeared in a punctate distribution. In addition, to highlight N2 activity in the embryonic periphery, the vector was injected into the left side of the embryo, resulting in left-side-dominant fluorescence at this stage. However, at later developmental stages, the majority of anterior epiblast-derived cells fluoresced. The red channel fluorescence data are shown in grayscale. The oval shape indicates a standard embryogenic region, N, the node position. **(A’)** Distribution of the brain portion (forebrain [FB], midbrain [MB], and hindbrain [B]), and spinal cord (SC) precursor positions in chicken embryos at st. 5 were determined by cell tracing (Yoshihi et al., 2022a), color-coded and superimposed on the N2 enhancer activity shown in **(A)**. **(B)** The epiblast cells at st. 5 were labeled randomly with EGFP fluorescence using the Supernova technique (Yoshihi et al., 2022a), and the trajectories of these cells were drawn up to late st.6 when the CNS precursor cells converged to the midline. The cyan line indicates the position of the anterior mesendoderm eliciting brain precursor convergence. The brain portion precursors migrated medio-anteriorly, as highlighted by green arrows. The SC precursors also converged to the midline but migrated medio-posteriorly, as indicated by the orange arrows. **(C)** Vector presentation of average cell migration rates and directions around the grid points on the epiblast of the same embryo as **(B)** at late st. 6. Grid intervals were 150 µm. Migrations of labeled cells positioned in a grid point-centered circle with a 150 µm radius were averaged over three consecutive frames (−10, 0, and +10 min). An arrow size equivalent to the 500 µm scale bar corresponds to the cell migration rate of 300 µm/h. Note the extensive posterior migration of the SC-forming cells at this stage. **(D)**. Distribution of the N2 enhancer-active cells of the same embryo as in **A** after establishing the CNS portions at st. 8, 18 h after st. 5 shown in **(A)**. The boundaries between the brain portions were assessed by morphological features, while the hindbrain/spinal cord boundary was determined as the position of one somite interval anterior to the first somite (Yoshihi et al., 2022a). All of the FB to the anterior SC were labeled by N2-mCherry. N2 enhancer is activated by Otx2 (Iwafuchi-Doi et al., 2012). The SC precursors were N2 active at st. 5 **(A)** but lacked *Otx2* expression in the posterior midline and forming SC (Chapman et al., 2002). Therefore, the labeling of SC cells by mCherry is due to the carryover of mCherry mRNA and protein after N2 enhancer shutoff; hence, SC labeling is weak. The N2 enhancer-positive cells outside the brain precursors in **A** developed into the head ectoderm (HE).

In a previous study, we determined the brain precursor map in the anterior epiblast of chicken embryos marked by *Sox2* N2 enhancer activation (Yoshihi et al., 2022a). In this study, we extended the analysis to anterior spinal cord (SC) precursors, also included in the N2 enhancer-marked anterior epiblast (Figures 1AA’). The fundamental technique was to randomly label the chicken embryo epiblast at st. 5 (∼20 h of incubation) by electroporating it with a Supernova vector cocktail at st. 4 (∼18 h of incubation) and to trace cells that contributed to the individual CNS portions at st. 8 (over ∼18 h). The previous analysis indicated that the epiblast regions for the developmental potential of the forebrain (FB), midbrain (MB), and hindbrain (HB) are arranged from anterior to posterior in the anterior epiblast. As shown in Figure 1A’, the SC precursors were mapped posterior to the HB precursors in the anterior epiblast.

The SC precursors migrated toward the midline and then posteriorly during early st. 5 to late st. 6, as shown by the trajectory of randomly labeled epiblast cells shown in Figure 1B, where the representative migration paths of the SC precursors are drawn in orange arrows. This migration path contrasts with the anterior migration of the brain precursors after convergence to the midline (green arrows). The vectors in Figure 1C show the average migration directions and velocities around the grid points at the end time point of the trajectories. Fast posterior migration of SC precursors along the midline (138 ± 39 (SD) µm/h) was observed.

The posterior migration of the anterior epiblast-derived SC precursors was reflected by the distribution of cells labeled by N2-activated mCherry, as shown in Figure 1D. The most posteriorly located SC precursors migrated over an axial distance of 2 mm; no more posterior labeling was observed (Figure 1D), indicating that the anterior epiblast-derived cells resided only in the anterior spinal cord. N2 enhancer activity depends on Otx2 activity (Iwafuchi-Doi et al., 2012; Uchikawa et al., 2017), but Otx2 expression is lost along the posterior migration route of SC precursors (Chapman et al., 2002); hence, the N2-indicating fluorescence declined but allowed tracing of the initially N2-positive cells in the spinal cord (Figure 1D, broken orange line).

In mouse embryos, the N2 enhancer-active region of the epiblast and forming neural tissues change analogously to the chicken embryos after E7 (Iwafuchi-Doi et al., 2011). In addition, analysis of stochastically labeled cell clones demonstrated clones comprising the brain and spinal cord subclones (Tzouanacou et al., 2009). Therefore, the mouse embryo anterior epiblast also serves as the origin of the CNS region spanning from the FB to the anterior SC.

### Establishment of NSC lines *in vitro* starting from EpiSCs under differential Wnt signal inputs

EpiSCs are maintained in culture media containing FGF2 and activin; the latter is an *in vitro* Nodal replacement that inhibits neural development (Camus et al., 2006). EpiSCs enter the neurogenetic process after removing activin/Nodal signaling and develop into neurons and glial cells (Brons et al., 2007; Tesar et al., 2007; Iwafuchi-Doi et al., 2012). A detailed analysis of the change in the gene expression profiles after activin removal indicated that the first day after activin removal from the culture gives rise to the transitory state between the epiblast state and the neurogenic state: the high Pou5f1 expression level is still maintained, yet neural primordium-characteristic Zeb2 (Sip1) expression (Chng et al., 2010) has started (Iwafuchi-Doi et al., 2012). Therefore, EpiSCs on the first day of activin removal mimic the transition state from the anterior epiblast to the neural primordium in embryos. To capture the neural primordial state immediately after the epiblast-to-neural-primordium transition, we placed activin-free EpiSCs in medium containing EGF and FGF2, which arrests neural development but maintain the cells as early-stage neural primordial cells. As discussed in the Introduction, establishing a stem cell line stabilizes a cell state that is transient in the normal developmental process. As the Wnt signal was a strong candidate agent specifying the anteroposterior regionality in the CNS primordia, we also manipulated Wnt signal levels on the NSC-generating EpiSCs in the following ways: 1) with the addition of Wnt signal antagonist XAV939 (XAV) at 10 µM to the culture medium (low Wnt condition) to obtain cell lines numbered with the prefix XN; 2) without the addition of Wnt antagonist/agonist allowing unconstrained action of cell-endogenous Wnt signaling (medium Wnt condition) for the cell lines with the prefix KN; and 3) with the addition of Wnt signal agonist CHIR at 2 µM (high Wnt condition) for the cell lines with the prefix CN. The cultures were passaged at an interval of 2–3 days on gelatin-coated dishes.

**FIGURE 2:**
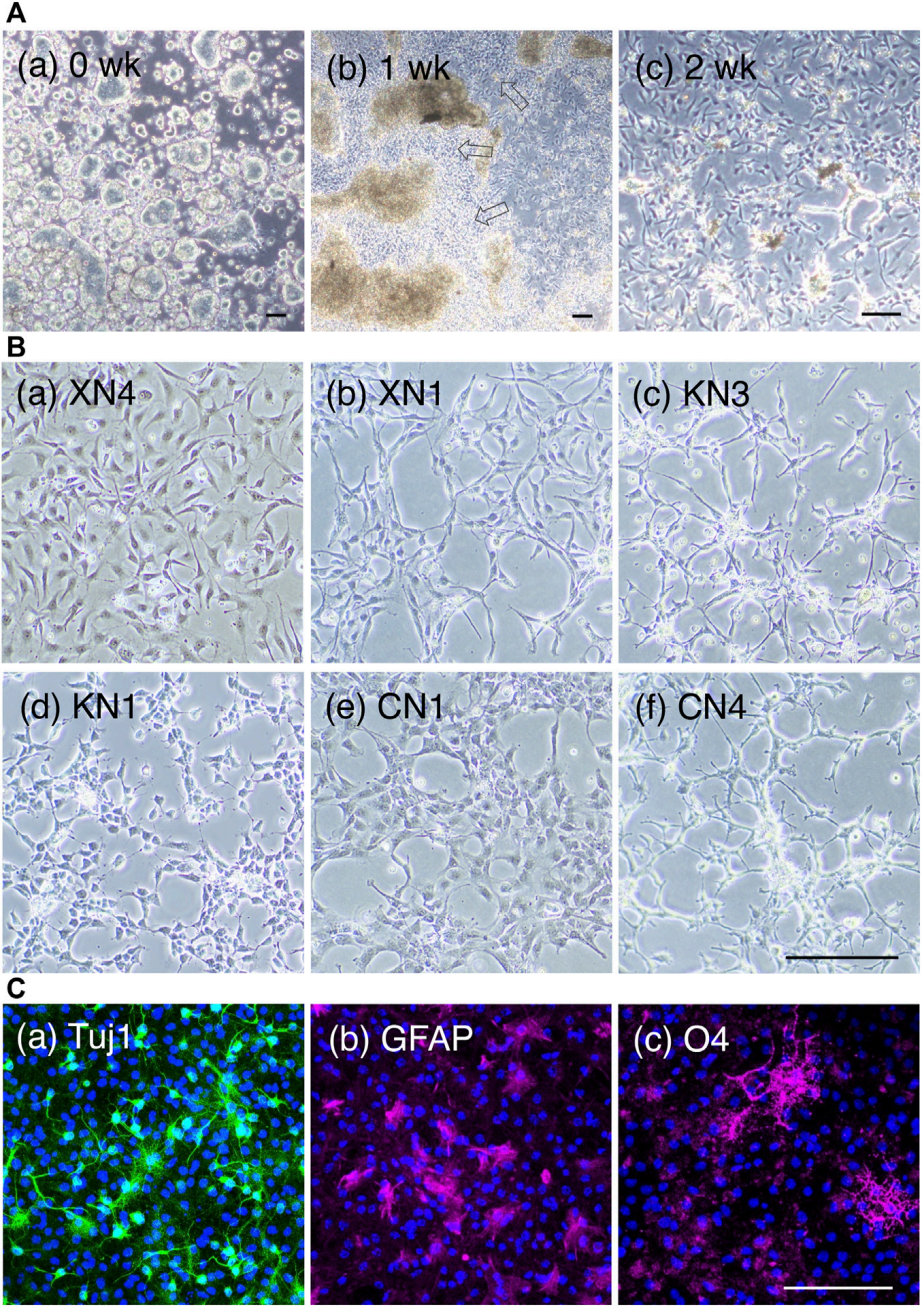
NSC lines derived from EpiSCs. **(A)** Intermediate culture states of NSC line development. Phase-contrasted bright-field images during NSC line development, showing the KN1 line. (a) One day after plating the EpiSCs on the gelatin-coated dish with the NSC culture medium. The cells attached to the culture dish formed large cell clusters. Bars, 100 µm. (b) One week after plating. NSC populations as outgrowths (open arrows) migrated out from the cell aggregates. (c) A 2-week culture displaying a homogeneous cell appearance. Bars, 100 µm. **(B)** Six cell lines were analyzed in depth in this study. The prefix “X” indicates a cell line derived with the addition of 10 µM XAV (Wnt antagonist) to the medium. “K,” without Wnt antagonist/agonist addition. “C,” with the addition of 2 µM CHIR (Wnt agonist). Scale bar, 100 µm. **(C)**. Neuronal and glial development of the NSC lines under respective differentiation medium, showing the case of the KN1 line. (a) Tuj1 immunofluorescence (green) showing neuronal cells after 3 days. (b) GFAP fluorescence (magenta) showing astrocytes after 5 days. (c) O4 antigen immunofluorescence (magenta) showing oligodendrocytes after 5 days. Nuclei (blue) were stained with 4′,6-diamidino-2-phenylindole (DAPI). The fraction of marker-positive cells in each three independent cultures were 21% ± 1% (SD) (Tuj1), 16% ± 1% (GFAP), and 28% ± 2% (O4). Scale bar, 100 µm.

In the first 4–5 days, cells developed as cell clumps loosely attached to the culture dish, and then cells adherent to the gelatin-coated dish started to migrate out of the cell clumps in a week; these cells proliferated well and prevailed in the cultures after serial passages (Figure 2A). The resultant cultures at 4 weeks appeared homogeneous in cell morphology in culture (Figure 2B). All these cell lines exhibited the characteristics of neural stem cell (NSC) lines (Supplementary Table S1), as detailed below.

We conducted NSC-producing cultures using different Wnt signal input levels in five series, as summarized in Supplementary Table S1, indicating the reproducibility of the NSC line derivation protocol. The six cell lines XN4, XN1, KN3, KN1, CN1, and CN4 were characterized in detail below, concerning gene expression profiles. The NSC lines were generated mostly using a mouse 129 line-derived EpiSC (Tesar et al., 2007). However, a BDF1-derived EpiSC line (Y62) used in series 2 similarly produced NSC lines, indicating that the derivation of an NSC line from EpiSCs using our protocol does not depend on the mouse origin of EpiSCs.

The cells in the NSC-generating culture multiplied similarly in the EGF- and FGF2-containing culture medium regardless of the addition of Wnt antagonist or agonist for ∼4 weeks. The resultant cell populations were morphologically homogeneous in culture but morphologically discernible between the NSC lines (Figure 2B). Notably, after 4 weeks of culture, the NSC lines started to display cell line-specific growth requirements, as summarized in Table 1. The cell lines XN1, CN1, and CN4 exhibited decreased cell growth under standard culture conditions, but the addition of the Rho kinase inhibitor Y27632 restored cell proliferation. In contrast, the addition of Y27632 inhibited the growth of XN4. The NSC lines also started to show different preferences concerning the culture substrate (gelatin or laminin) for their growth. Furthermore, the KN3 line was found to be sensitive to Accutase treatment.

**TABLE 1:** Effects of Y27632, Accutase treatment, and culture substratum on the NSC lines.

NSC line	Effect of Y27632 on cell growth	Cell damage by accutase treatment	Substrate preference for growth: Laminin vs. gelatin
XN4	Inhibition	Minimal	Gelatin
XN1	Promotion	Minimal	Laminin
KN3	No significant effect	Severe	Equal
KN1	No significant effect	Minimal	Laminin
CN1	Promotion	Minimal	Laminin
CN4	Promotion	Minimal	Gelatin

In summary, the EpiSC-derived NSC lines acquired each unique trait after 4 weeks of culture: responses to the Rho kinase inhibitor, the culture substrate, and Accutase treatment. This observation may indicate that the cell populations reach the state of mature and stable cell lines with specific growth requirements. A feature shared by all six NSC lines was the dependence of their cell growth on the local cell density; in a rough estimation, a local cell density (not the average of a culture) of 400 cells/mm^2^ was required for their growth.

These cell lines were proven to be NSC lines that could develop into neurons expressing ßIII tubulin (Tuj1) and glial cells (astrocytes expressing glial fibrillary acidic protein (GFAP) and oligodendrocytes expressing the O4 antigen) under their differentiation conditions (Figure 2C, Supplementary Table S1).

### Transcriptome analysis allocating the NSC lines along the A/P axis of the embryonic CNS

**FIGURE 3:**
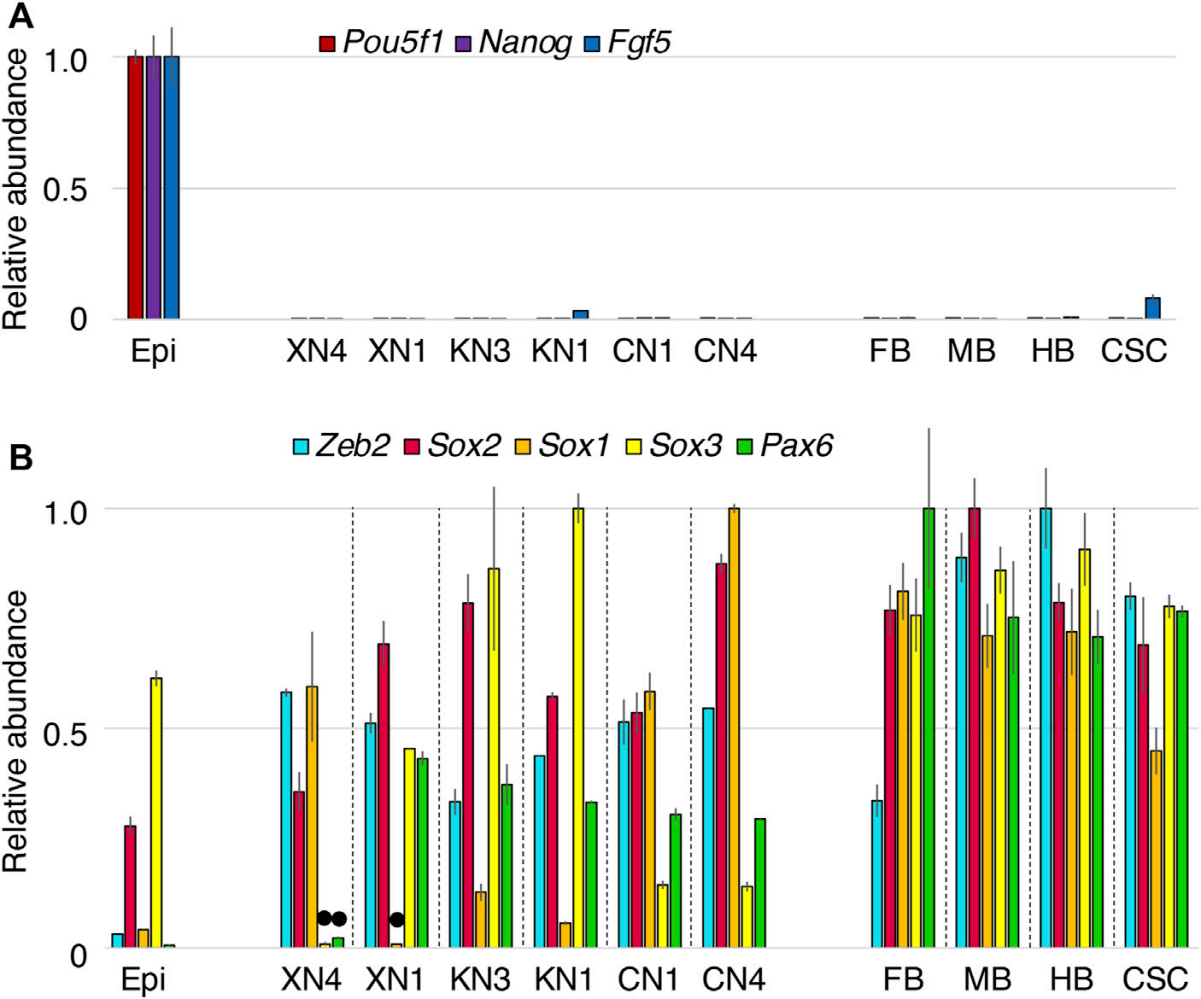
Expression levels of neural-characteristic genes in the NSC lines compared with the E10.5 mouse CNS portions. The microarray signal intensity data of biological replicates were compared for the six NSC lines, EpiSCs (Epi), forebrain (FB), midbrain (MB), hindbrain, and cervical level spinal cord (CSC). The highest expression level among the specimens was referred to as 1.0 for each gene transcript. Error bars indicate the range of variations in the detected signal intensity in duplicate samples. **(A)** The epiblast-characteristic genes Pou5f1, Nanog, and Fgf5 were downregulated in all NSC lines. **(B)** Expression levels of the neural-characteristic TF genes Zeb2, Sox2, Sox1, Sox3, and Pax6. The expression levels of these genes in the NSCs were in the same order as those in E10.5 CNS, except for the low Sox3 and Pax6 levels in the XN4 line and the low Sox1 level in the XN1 line indicated by black dots. Sox2 and Sox3 are also expressed in epiblasts (Uchikawa et al., 2011) and EpiSCs (Iwafuchi-Doi et al., 2012). Sox2 (most prevailing expression) and Sox1/3 (more regionally restricted expression) belong to the B1 Sox class genes and are functionally redundant (Uchikawa et al., 2011; Kamachi and Kondoh, 2013).

The transcriptome profiles of the NSC lines derived from EpiSCs under different Wnt signal inputs were characterized using microarrays. The expression of genes was also compared with the FB, MB, HB, and cervical SC (anterior to the forelimb buds) of E10.5 mouse embryos concerning the patterns and expression levels (Figure 3).

Analysis of landmark gene transcripts characteristic of EpiSCs and NSCs indicated overt differences between these cells. EpiSC-characteristic genes, including Pou5f1, Nanog, and Fgf5, were all downregulated in all six NSC lines (Figure 3A). In contrast, the NSC-characteristic genes itemized below were activated to levels comparable to those in the E10.5 CNS (Figure 3B). Neural progenitor-characteristic TF transcripts *Zeb2* *(Sip1)* (Chng et al., 2010; Dang et al., 2012; Hegarty et al., 2015) and *Sox2*, the primary B1 *Sox* gene (Uchikawa et al., 2011; Kamachi and Kondoh, 2013), were expressed at significant levels. At least one of the subsidiary B1 *Sox* genes, *Sox3* and *Sox1*, was strongly expressed; Sox3 expression was low in the XN4 cells, while Sox1 *expression* was low in the XN1 cells. Pax6 expression was also high in the NSC lines except for the XN4 cells. The variations in Sox3/1 and Pax6 expression presumably reflect the anteroposterior regional specification of individual NSC lines, which is presented below.

**FIGURE 4:**
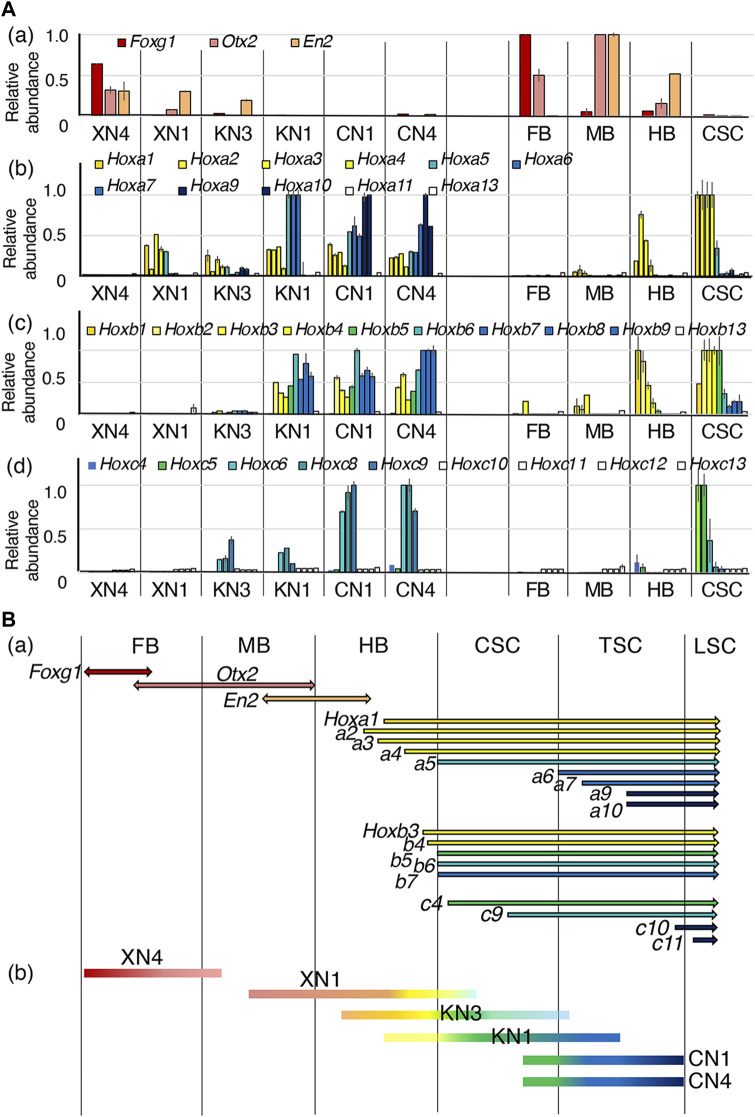
Assessment of the anteroposterior regional coverages by the NSC lines. **(A)** Expression profiles of brain-specific TFs and the *Hoxa*, *Hoxb,* and *Hoxc* genes expressed in the six NSC lines and E10.5 mouse embryo FB, MB, HB, and CSC. The microarray signal intensity data of biological replicates were compared. The highest expression level of a gene in the mouse embryo CNS portions was referred to as 1. Error bars indicate the range of variations in the detected signal intensity in the duplicate samples. (a) Expression profiles of brain-specific TFs: *Foxg1* in the FB (Shimamura et al., 1997; Martynoga et al., 2005), *Otx2* in the FB and MB (Simeone et al., 1992; Ang et al., 1994), and *En2* in the posterior MB and anterior HB (Davis and Joyner, 1988). (b–d) Expression profiles of *Hoxa*, *Hoxb*, and *Hoxc* genes in individual NSC lines. **(B)** (a) The ranges of expression of the genes across the CNS domains. The expression profiles are indicated according to Carpenter (2002), E10.5 *in situ* hybridization data in the EMAGE gene expression database (http://www.emouseatlas.org/emage/) (Richardson et al., 2014), and the data in this study. (b) The anteroposterior regions covered by NSC lines assessed by the TF expression profiles.

To allocate the EpiSC-derived NSC lines along the anteroposterior axis of the embryonic CNS, the expression levels of brain region-characteristic TF genes (*Foxg1*, the forebrain (FB); Otx2, the FB and midbrain (MB); and En2, the MB and hindbrain (HB)), as well as the Hox genes expressed posterior from the HB, were analyzed. To graphically represent the data, the highest expression level among the ten sample groups (six NSC lines and the four brain portions) measured in the microarray analysis was referred to as 1.0 (Figure 4). Overall, the TF genes mentioned above were expressed in the NSC lines at the level ranges observed in the E10.5 mouse CNS.

The expression profiles of the brain-characteristic genes (Figure 4Aa) showed that XN4 expressed *Foxg1* at a level comparable to that of the embryonic FB and low levels of *Otx2* and *En2* (MB/HB) but no *Hox* genes (posterior from HB), indicating that the XN4 line primarily showed FB regionality.

Other NSC lines expressed various *Hox* genes. The regionality codes of *Hox* gene expression from the hindbrain to the SC around E11 were determined first based on the data by Carpenter (2002), the whole-mount *in situ* hybridization data of E10.5 mouse embryos in the EMAGE database, and the CSC data here, as summarized in Figure 4Ba. The data for the expression of *Hoxa*, *Hoxb*, and *Hoxc* genes in the NSC lines are shown in Figures 4Ab–d, which indicated that *Hoxa1*, *a2*, *a3*, and *a4*, as well as *Hoxb1*, *b2*, *b3*, and *b4*, collectively called the HB class in this study, have the anterior expression boundary in the HB domain. Likewise, *Hoxa5*, *Hoxb5*, and *Hoxb6* (the CSC class) have their anterior expression boundary in the cervical spinal cord (CSC, anterior to the forelimb bud). *Hoxa6*, *a7*, *a9*, and *a10* (the TSC class) appeared to be expressed posterior to the CSC in the thoracic spinal cord (TSC). The anterior expression limits for *Hoxb7*, *b8*, and *b9* (also the TSC class) are presumably around the CSC and TSC junction, as a low level of expression was measured in the CSC specimens (Figure 4Ba). *Hoxa13* or *Hoxb13* characteristic of the caudal SC were not expressed in any of the specimens. *Hoxa13* or *Hoxb13,* characteristic of the caudal SC, were not expressed in any samples. Important information concerning the thoracic and lumber levels is that *Hoxc9* expressions present in the TSC, while Hoxc10 is expressed posterior to the lumbar SC (LSC).

Based on the above criteria, anteroposterior regional coverages of NSC lines expressing *Hox* genes were tentatively assessed (Figure 4Bb).

1) The XN1 line expressed *Otx2*, *En2*, HB class *Hox* genes, and *Hoxa5* but not *Hoxb5*, indicating its regional coverage from MB to the anterior CSC. (The observation places the anterior boundary of *Hoxa5* expression anterior to that of *Hoxb5.*)
2) The KN3 line expressed *En2*, *Hox* genes of the HB class, and at low levels CSC and TSC classes, suggesting its primary coverage starting from the HB domain with a posterior extension.
3) The KN1 line was characterized by the absence of brain-characteristic gene expression and strong expression of HB and CSC class *Hox* genes, *Hoxa6-7* and *Hoxb7-9*, but without expression of *Hoxa9*-*10*, determining its regionality coverage from the posterior HB/CSC to the anterior TSC.
4) The CN1 and CN4 lines were similar concerning the anteroposterior regional identities, expressing the *Hox* genes of HB, CSC, and TSC classes, including *Hoxa9*-*10.* Remarkably, CN1 and CN4 expressed *Hoxc9* but not *Hoxc10*, indicating that the posterior limit of regional coverage of these NSC lines is at the thoracic level.

Significantly, the domain coverages by the NSC lines were ordered from anterior to posterior according to the Wnt signal inputs. Very similar TF expression profiles indicative of posterior positioning of CN1 and CN4 that developed in the presence of the Wnt signal agonist CHIR at 2 μM confirm this notion. The lines KN3 and KN1 developed solely under cell-endogenous Wnt signaling but differed slightly in regional coverage. This difference can be accounted for by the fluctuation of endogenous Wnt signal levels in the starting EpiSCs, which was reported in human ESCs resembling EpiSCs (Moya et al., 2014).

Whereas the XN4 line expressed *Foxg1*, a hallmark of the forebrain precursor, XN1 line did not express *Foxg1* but expressed anterior *Hox* genes and midbrain-centered characteristics. The XN4 line was produced using 10 µM XAV from supplier B (Supplementary Table S1), but the XN1 line was generated using 10 µM XAV from supplier A, which developed crystalline precipitates during the culture incubation (Supplementary Figure S1; Supplementary Table S1). This crystal precipitation must have decreased the effective concentration of XAV from supplier A, as discussed in Supplementary Figure S1 legend. This difference in the effective XAV concentrations accounts for the different anteroposterior regionalities between the XN4 and XN1 lines.

In summary, all six NSC lines derived from the EpiSCs under different levels of Wnt signal input displayed distinct anteroposterior regionalities following the Wnt signal strengths, from the FB (the lowest Wnt signal input) to the TSC (the highest Wnt signal input). However, their regionalities were broad, covering more than one CNS domain (Figure 4Bb), which was investigated using single-cell transcriptome analysis presented below.

### The NSC lines that developed without exogenous signaling other than Wnt signal manipulation gained ambiguous dorsoventral identities

**FIGURE 5:**
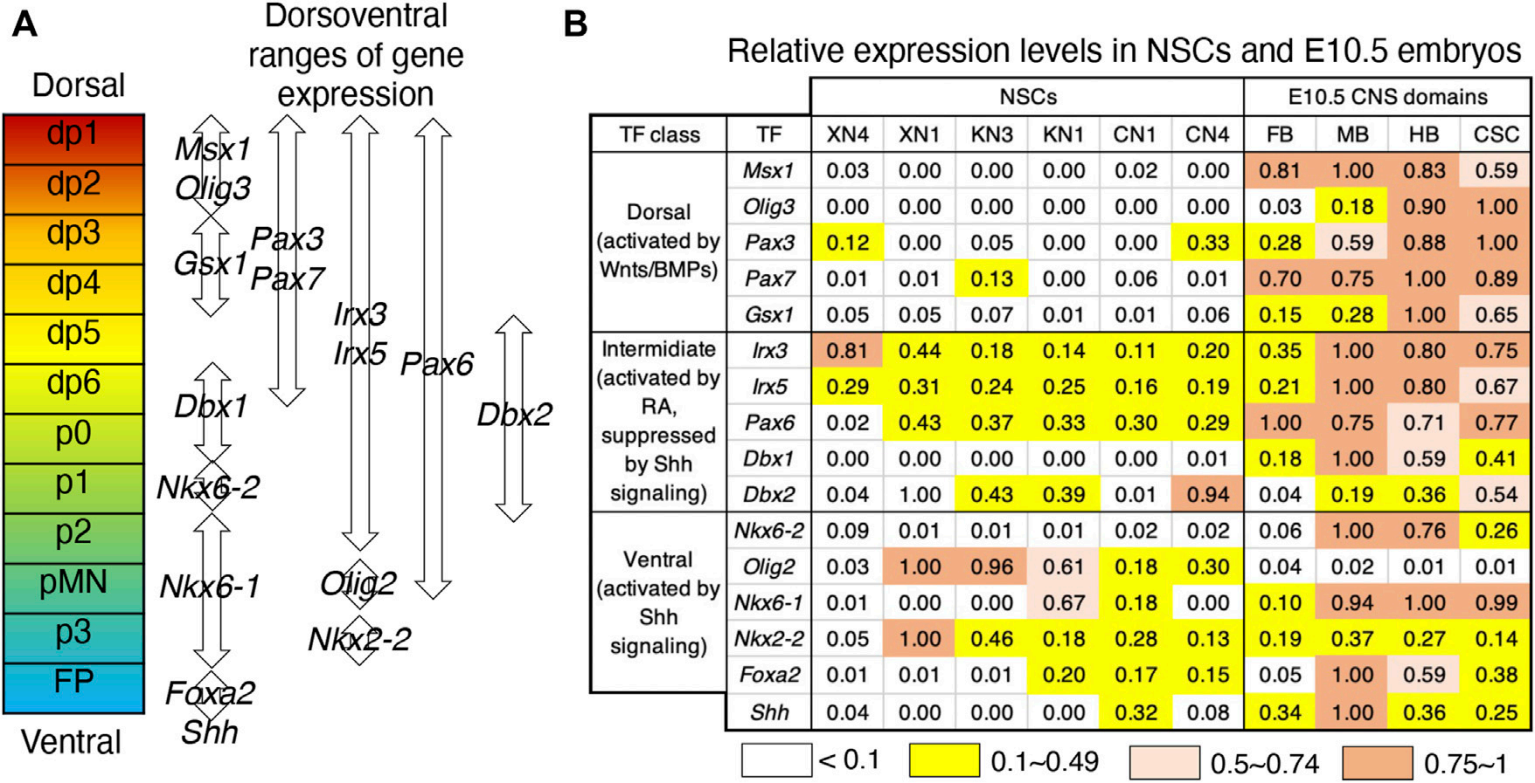
Ambiguous dorsoventral identities of the NSC lines. **(A)** Dorsoventrally arranged neural progenitor domains of the neural tube defined by overlapping TF expression patterns (Sagner and Briscoe, 2019). **(B)** Relative expression levels of the TF genes specifying the dorsoventral neural tube domains in the NSC lines and E10.5 embryonic CNS domains. The highest microarray signal intensity in the dataset was referred to as 1.0, and the expression level groups are color-coded as indicated at the bottom. The TF genes are classified into dorsal, intermediate, and ventral classes according to their responses to exogenous signaling (Andrews et al., 2019).

In the mouse neural tube, dorsoventral regionalization also occurs from E8 to E11 during embryogenesis under the influence of dorsal-character-inducing Wnt/BMP signals and ventral-character-inducing sonic hedgehog (Shh) signals (Andrews et al., 2019; Sagner and Briscoe, 2019). Dorsoventrally arranged neural tube subregions are marked by differential TF expression (Figure 5A). In contrast to the anteroposterior regionalities given to the NSC lines, these lines did not develop clear dorsoventral identities (Figure 5B). The only commonly expressed TF genes were members of an intermediate class (*Irx3* and *Irx5*), covering the wide dorsal region, but they were generally unaccompanied by dorsal neural progenitor-characteristic *Pax3/Pax7* expression (Gard et al., 2017). The ventral p3 domain-characteristic *Nkx2-2* and *Nkx6-1* are activated in response to Shh signaling in spinal cord neural progenitors (Andrews et al., 2019; Sagner and Briscoe, 2019), but the NSC lines, e.g., XN1, expressed *Nkx2-2* without concurrent *Nkx6-1* or correlation with endogenous Shh expression levels, ruling out the model that *Nkx2-2* expression represents ventral CNS specification. Most notably, the TF gene expression analysis showed no dorsal or ventral bias in response to the Wnt signaling levels during the cell line derivation, although the Wnt signal is one of the primary cues to activate TF genes of the dorsal class (Muroyama et al., 2002). This observation confirmed that the NSC lines from activin-free EpiSCs represent an earlier developmental period than the dorsoventral specification of neural tubes.

### Process of establishing the NSC characteristics and regional specificities

**FIGURE 6:**
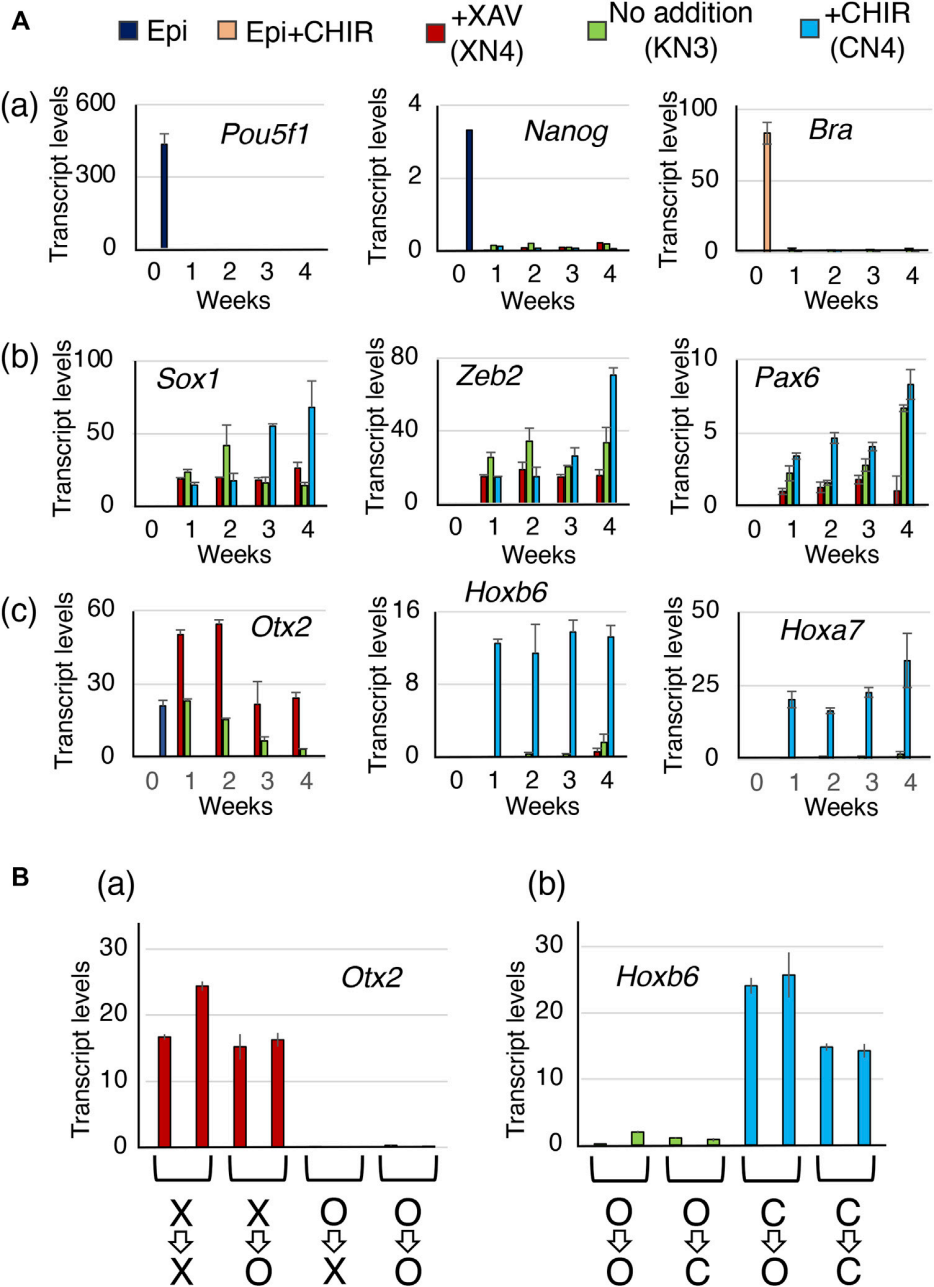
The temporal expression profiles of the TF genes during the development of the XN4, KN3, and CN4 NSC lines. **(A)** Gene expression levels were measured at 1-week intervals, comparing EpiSCs (0 weeks) and the NSC intermediates under culture conditions with 10 µM XAV, no addition, and 2 µM CHIR. Transcript levels were determined using RT‒qPCR and indicated as 1000 × relative to the *Gapdh* level. The averages of four measurements (two biological replicates with two technical replicates) and standard errors are shown. (a) Expression of the epiblast-specific genes *Pou5f1* and *Nanog* during the derivation of the NSC lines and the mesoderm-characteristic *Bra* gene activated by 2 µM CHIR in EpiSCs. They were downregulated after 1 week in the NSC-developing culture conditions. (b) Expression of the NSC-characteristic genes *Sox1*, *Zeb2*, and *Pax6*. All these genes were activated after 1 week of culture. (c) Expression of the CNS domain-characteristic genes *Otx2* (FB and MB), *Hoxb6* (SC), and *Hoxa7* (SC). Only in the CHIR addition condition, giving rise to the CN4 line, was the *Otx2* gene quickly shut off, whereas the *Hoxb6*/*Hoxa7* genes were activated in 1 week. In the XAV-addition condition, giving rise to the XN4 line, *Otx2* expression was maintained. Under conditions without exogenous manipulation of Wnt signaling to derive the KN3 line, *Otx2* expression diminished gradually toward the 4-week point. The averages of four measurements (two biological replicates with two technical replicates) and standard errors are shown. **(B)** Evidence that the CNS regional characteristics were already established in 3 weeks of NSC-generating cultures. Subcultures were prepared from the 3-week cultures under different Wnt signal-manipulating conditions. The subcultures were placed in other culture conditions for one additional week (the equivalent of 4-week main culture): X=>X, keeping XAV addition; X=>O, from XAV addition to no addition; O=>X, from no addition to XAV addition; O=>O, keeping no addition; O=>C, from no addition to CHIR addition; C=>O, from CHIR addition to no addition; C=>C, keeping CHIR addition. Two subcultures were prepared for each condition, and the averages and data ranges of the technical replicates are shown. The cells maintained the *Otx2* or *Hoxb6* expression statuses of the 3-week cultures even after changing the culture conditions.

The changes in TF gene expression were evaluated using RT‒qPCR at 1-week intervals during the development of the XN4, KN3, and CN4 lines under the following conditions: with the addition of 10 mM XAV, without any additives, or with the addition of 2 mM CHIR. The aim was to investigate how the NSC characteristics converge after 4 weeks of NSC line derivation (Figure 6).

The EpiSC-characteristic genes Pou5f1 and Nanog were quickly inactivated in a week in all three NSC culture conditions (Figure 6Aa). Although Bra is strongly activated in EpiSCs in the activin-FGF2 medium with 2 mM CHIR, *Bra* was not activated in the NSC-developing culture conditions, even during the CN4 line derivation with 2 mM CHIR (Figure 6Aa). NMPs characterized by the coexpression of *Bra* and *Sox2* around the time of dichotomic fate choice (Figure 6Aa) (Kondoh and Takemoto, 2012), although the possibility of a transient activation *Bra* during the sampling intervals was not formally excluded. This notion is consistent with the observation that the contribution of anterior epiblast-derived cells to the spinal cord was limited to the anterior part of the spinal cord (Figure 1D; Figure 4Ad).

The characteristic neural genes, *Sox1*, *Zeb2*, and *Pax6*, which were silent in the EpiSCs, were activated in 1 week in all three culture conditions (Figure 6Ab). The low-Pax6 expression of the XN4 line was observed from the earliest time point.

*Hoxb6* and *Hoxa7* genes representing the thoracic levels of the spinal cord were quickly activated by 1 week under the high Wnt condition, and the expression level was maintained up to the 4-week point when the CN4 line was established (Figure 6Ac). The level of *Otx2* expression was sustained under the low Wnt condition (XAV addition) that gave rise to the XN4 line. In contrast, under the high Wnt condition (CHIR addition), *Otx2* expression was turned off from the beginning despite being expressed in the EpiSCs. The case of the KN3 line was intermediate.

During the culture process to produce the NSC lines, the cells were passaged and maintained under the same culture conditions with or without the addition of a Wnt antagonist (XAV) or Wnt agonist (CHIR). However, the anteroposterior regional specificities were created during the development of the NSC lines rather than in response to the culture conditions after the NSC line establishment.

After 3 weeks of culture in the presence of Wnt antagonist, Wnt agonist, or no addition, subcultures were prepared to compare the effects of different culture conditions on Wnt signal levels. 1) Four subcultures of cells developed in the XAV-containing medium were prepared; a pair was maintained in the medium with XAV (X=>X) and the second pair without XAV (X=>O). The subcultures were separately maintained over a week with two to three passages. 2) Similarly, six subcultures of cells that had developed in the absence of Wnt antagonist/agonist were cultured pairwise in media with XAV (O=>X), without addition (O=>O), or with CHIR (O=>C). 3) Four subcultures of cells developed in the CHIR-containing medium were placed pairwise in the agonist-free medium (C=>O) or the CHIR-containing medium (C=>C). After a week of subculturing (i.e., the same chronological point as the establishment of the XN4, KN3, and CN4 lines), the sublines were examined for the expression of an anterior-characteristic gene *Otx2* and for a posterior characteristic gene *Hoxb6*.

These cell subcultures under conditions of altered Wnt signal inputs did not affect the anteroposterior characteristics of the genes. All X=>X and X=>O cultures maintained a level of *Otx2* expression, whereas *Otx2* expression remained very low in O=>X and O=>O cultures (Figure 6Ba). Similarly, all C=>C and C=>O cultures displayed high levels of *Hoxb6* expression, while *Hoxb6* expression in O=>C and O=>O cultures remained low (Figure 6Bb).

These observations indicate that the anterior and posterior regional characteristics imposed by the Wnt antagonist and Wnt agonist in the anterior epiblast-derived neural primordia had already developed during a week of culture. The stabilized anteroposterior and other characteristics of the NSC lines developed during the 4-week culture period.

### Single-cell transcriptome analysis of the NSC lines

**FIGURE 7:**
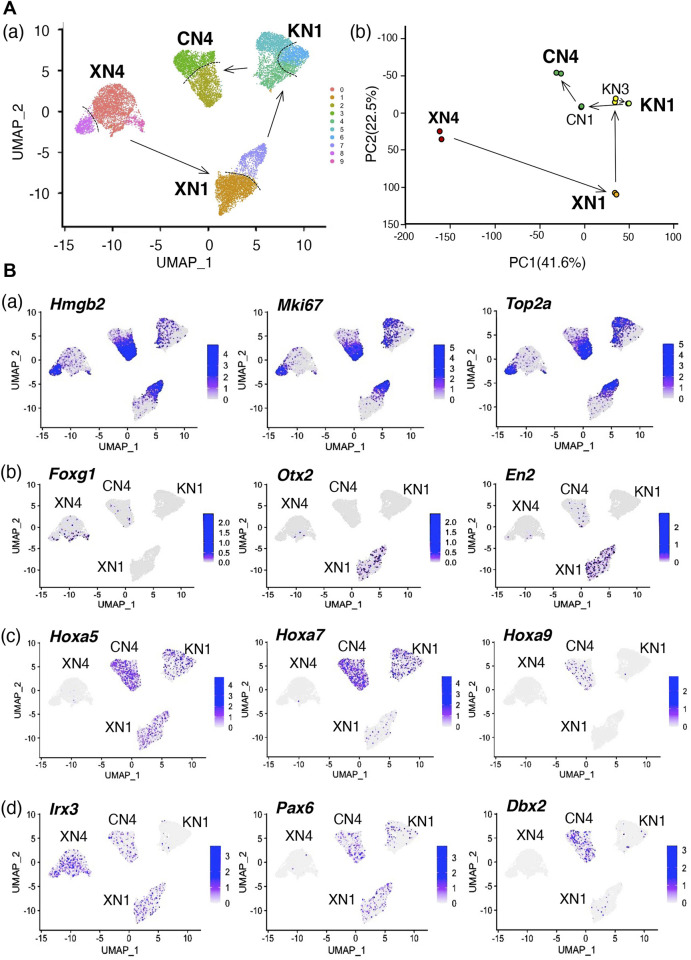
Single-cell transcriptome data in UMAP plots show that the NSC lines represent inseparable cell groups sharing each unique feature. **(A)** Transcriptome-based relationships among the NSC lines. (a) UMAP clustering of the single-cell transcriptome data of the XN4, XN1, KN1, and CN4 NSC lines. XN3 line was not included in the analysis, as the XN3 cells did not withstand dissociation into single cells as reported in Table 1. The arrows indicate the anteroposterior order of the NSC lines, as determined in Figure 4. The cells of individual NSC lines form tight clusters, divided into two main subclusters indicated by the dotted lines. As shown in (B) (a), these subdivisions represent different cell cycle phases. (b) Principal component (PC) analysis of the bulk transcriptome data of the six NSC lines using microarrays. Separate dots represent the data of replicate samples. The arrows indicate the anteroposterior order of the NSC lines. Enlarged characters indicate the NSC lines analyzed in Panel (a). The overall arrangement of the NSC line data in the PC1-PC2 projection is similar to the UMAP projections shown in Panel (a), indicating the consistency between the bulk and single-cell transcriptome analysis. **(B)** Various features of the NSC lines are displayed on the UMAP plot of cell clusters. The color scales are presented in a 2-based logarithm. Note that the gene expression-detected cells were distributed randomly within an NSC cluster in Panels (b–d). (a) The distribution of the G2-M phase-characteristic gene transcripts, *Hmgb2*, *Mki67*, and *Top2a*, concentrated in cells of one of the subclusters of the NSC clusters. (b) Distributions of brain domain-characteristic transcripts. *Foxg1*, characteristic of FB, was detected in the XN4 cluster, whereas the expression of *Otx2* (FB and MB) and *En2* (MB and HB) was conspicuous in the XN1 cluster. (c) Expression of an array of *Hoxa* genes with the anterior boundary of the expression domain in the CNS moving posteriorly with increasing gene numbers. *Hoxa5* expression was found in the XN1, KN1, and CN4 clusters, *Hoxa7* in the KN1 and CN4 clusters, and *Hoxa9* in CN4. (d) Expression of TF genes that are expressed in a dorsoventrally organized domain of the embryonic neural tube (see Figure 5). *Irx3* transcripts were distributed in cells of the XN4, XN1, and CN4 clusters, *Pax6* in the XN1, KN1, and CN4 clusters, and *Dbx2* in the CN4 cluster.

The microarray data for the cell groups of the NSC lines presented in Figure 4 indicated that each NSC line has a relatively broad anteroposterior regional coverage. For instance, the XN1 line expressed the *Otx2*, *En2*, and *Hoxa* genes of HB regionality, covering the MB and HB regions. It is critical to determine whether the broad anteroposterior specificity is intrinsic to individual cells of the cell line or whether the cell lines are composites of separable subpopulations with narrower anteroposterior specificities. To address this issue, we performed single-cell transcriptome analysis (single-cell RNA-seq) of the four cell lines XN4, XN1, KN1, and CN4 (Figure 7). The uniform manifold approximation and projection (UMAP) of the single-cell transcriptome data indicated that individual cell lines formed tight clusters. The arrangement of the four clusters representing the NSC lines, showing the relative distances among the cell states (Figure 7Aa), was consistent with the primary component analysis (PC1 and PC2) of the microarray data of the bulk transcripts (Figure 7Ab), indicating that the general features of the bulk transcriptomes are reflected in the single-cell transcriptomes.

Each transcriptome cluster representing an NSC line was divided into two (or three at most) subclusters, indicated by the broken lines separating the subclusters in Figure 7Aa. However, these subclusters did not represent separable cell populations but the cells in different cell cycle phases. As shown in Figure 7Ba, one of the subclusters was filled with cells expressing *Hmgb2*, *Mki67*, and *Top2a*, which are characteristic of cells in the G2 to M phases (Pallier et al., 2003; Daniloski et al., 2019; Uxa et al., 2021).

Concerning the brain region characteristic genes (Figure 7Bb), the XN4 cluster contained *Foxg1*-expressing cells, and the XN1 cluster had cells with *Otx2* expression and *En2* expression. The cells where these transcripts were detected were distributed more or less randomly in the NSC clusters, indicating that the anteroposterior regionality does not divide the NSC clusters. Similarly, *Hoxa5*-expressing cells were abundant in the XN1, KN1, and CN4 clusters, *Hoxa7*-expressing cells in the KN1 and CN4 clusters, and *Hoxa9* specifically in the CN4 cluster, following the more posterior activation of *Hoxa* genes with larger numbers. Again, the cells with the detection of these *Hox* transcripts were distributed randomly in an NSC cluster.

Similarly, the cells with genes normally involved in the dorsoventral regionalization of the neural tube were distributed with the NSC clusters as units. *Irx3* transcript-captured cells were abundant in the XN4, XN1, and CN4 clusters, *Pax6*-captured cells in the XN1, KN1, and CN4 clusters, and *Dbx2*-captured cells in the CN4 cluster.

The random distribution of the cells with expression of TF genes for different anteroposterior characters in an NSC line cluster opposed the model that the NSC lines were composites of distinct subpopulations bearing narrow regionalities.

### Single cells express multiple genes representing a range of regional specificities

**FIGURE 8:**
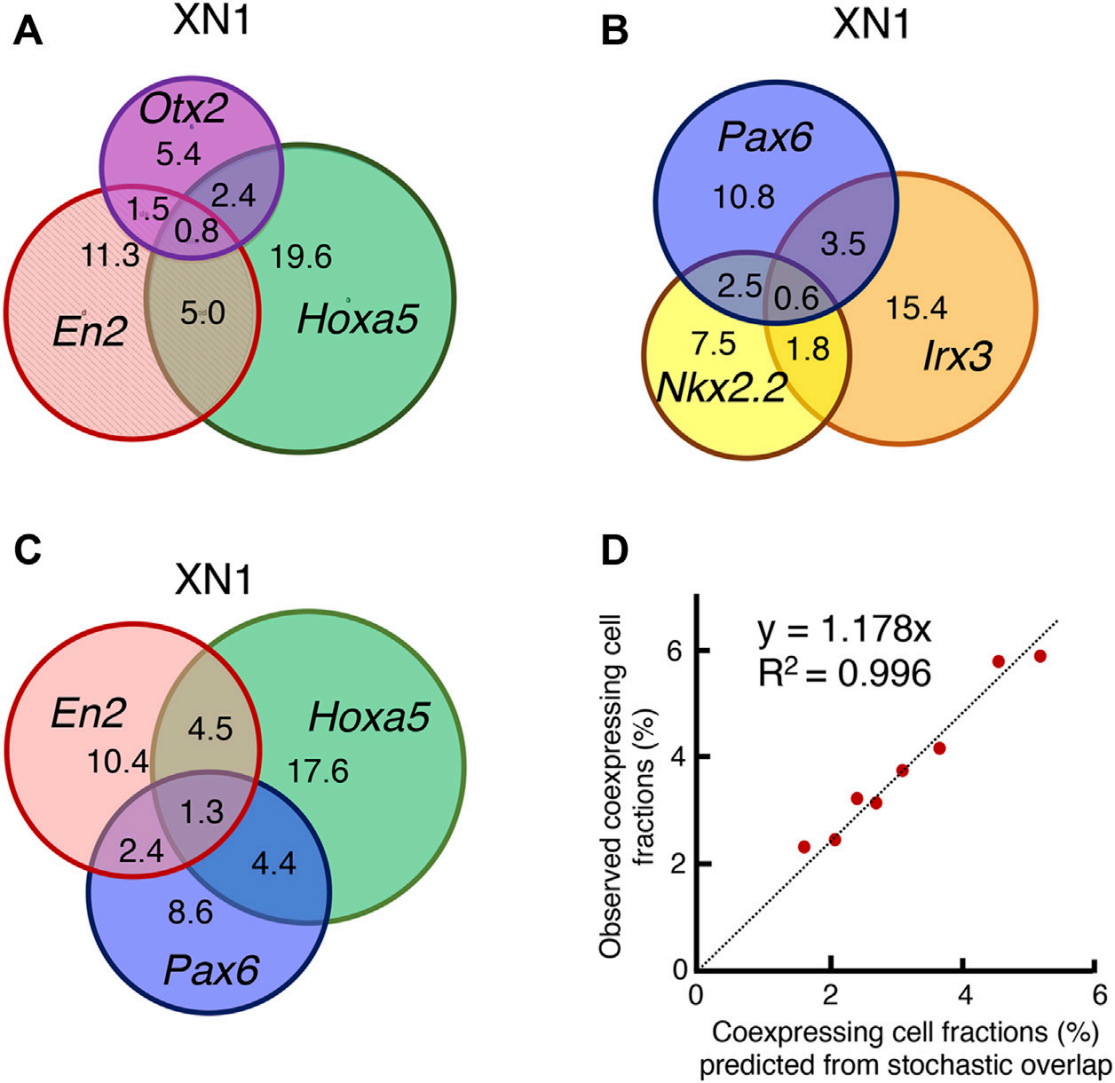
Analysis of transcripts in individual cells. **(A)** Among the XN1 cells, the fraction of cells in which the transcripts of *Otx2*, *En2*, and *Hoxa5* were found individually or in combination were scored and presented in an area-proportional Venn diagram. These three genes were expressed without correlation. A substantial fraction of cells coexpressed *Otx2* and *Hoxa5*, which does not occur in the CNS after E10.5 (Figure 4). **(B)** Similar analysis of the TF genes in XN1 cells involved in the dorsoventral specification of the embryos after E10.5. These genes were expressed without mutual correlations. **(C)** Similar analysis comparing the expression of anteroposterior specification of the CNS primordia (*En2* and *Hoxa5*) and *Pax6* involved in dorsoventral specification. These TF genes were also expressed without mutual correlations. **(D)** Testing the model of the stochastic and independent expression of these TF genes. The model predicts that an overlap of the expression of two TF genes occurs in proportion to the multiplication products of the frequency of one TF expression. The predicted and observed frequencies of the coexpression of the two TF genes were aligned on the regression Line y = 1.178x with *R* ^2^ = 0.996, supporting the model. The preferential representation of cells with higher global capture rates for gene transcripts accounts for a slightly higher value of the observed frequencies.

To clarify whether single cells simultaneously express genes representing different regional specificities, transcriptomes in individual cells were analyzed. The results are presented in Figure 8 using area-proportional Venn diagrams. The numerals indicate the percent of cells where the transcript was detected.

Figure 8A illustrates the expression and coexpression rates of *Otx2*, *En2*, and *Hoxa5* in the XN1 cell population. A substantial fraction of the cell population expressed two or three genes together without specific indication of coexpression preferences. Particularly noteworthy is the presence of cells expressing both *Otx2* and *Hoxa5* (3.2% among 45.3% of cells with transcript capturing of one of these TFs), with a cell population size larger than those with *Otx2*-*En2* coexpression (2.3%). Cells expressing *Otx2*, *En2*, and *Hoxa5* (0.8%) were also identified. The situation was analogous concerning the expression of TF genes, which are normally expressed according to dorsoventral regionalities (Figure 8B), and among the genes for anteroposterior regionalities (*En2* and *Hoxa5*) and dorsoventral regionality gene *Pax6* (Figure 8C). As the coexpression rates of two TF genes appeared to reflect the fraction of cells expressing individual TFgenes, we tested the model in which individual TF genes were expressed stochastically. Namely, if TF genes P and Q are expressed in the fractions p and q of the cell population, the coexpression frequency for P and Q is predicted to be p multiplied by q. Figure 8D compares predicted and observed TF gene coexpression frequency using data shown in Figures 8A–C. The data were on the regression Line y = 1.178x through the axis origin with *R* ^2^ = 0.996. The nature of single-cell transcriptome analysis accounts for the 17.8% increase in the rate of observed coexpression. The sequence read depths are highly variable among the cells. As the detection rate of coexpressed gene transcripts is higher in more deeply read cells, the observed coexpression rate becomes higher than the predicted rate based on the averaged detection rates of single TF genes. The same scenario also applies to the TF genes representing anteroposterior regionalities in the XN4, KN1, and CN4 NSC lines (Supplementary Figure S2).

Taken together, the transcriptome analysis at the single-cell level (Figures 7, 8) indicated that each NSC line consisted of cells bearing a range of anteroposterior specificities, which accounted for the microarray expression profile of the NSC lines, as displayed in Figure 4. The analysis identified cells expressing two or three such genes simultaneously and cells expressing single genes representing different regional characteristics. The statistical analysis indicated that the coexpression of multiple genes occurred stochastically, without a preference for anteromedial or medioposterior regional preferences.

## Discussion

### A strategy for modeling an early stage of anteroposterior specification in neural primordia

A study using chicken embryos for brain portion development (Yoshihi et al., 2022a; Yoshihi et al., 2022b) and spinal cord development (Figure 1) indicated that anteroposterior regional specificities are grossly specified at the epiblast stage, before neural plate development. In chicken embryogenesis, the CNS precursors in the Otx2-expressing, and hence N2-enhancer active, anterior epiblast first converge to the midline, as do other cells involved in gastrulation through the primitive streak. In contrast, in mouse embryos, the cell convergence process is missing; anterior epiblast cells develop *in situ* into brain tissues (Cajal et al., 2012), and only primitive streak-abutting cells ingress into the axial mesoderm compartment, at least during the early stages of gastrulation (Williams et al., 2012). Nevertheless Metzis et al. (2018), using an ESC-derived neurogenesis *in vitro* model, showed that the chromatin accessibility pattern of anteroposterior regional characteristics of the CNS primordia is established before neurogenesis initiates, indicating that early-stage anteroposterior specification is common between chicken and mouse embryogenesis. The model of the brain portions and the anterior spinal cord arising from a consecutive epiblast domain in mouse embryos without the involvement of NMPs, as in chicken embryos, has been supported by the presence of randomly labeled clones encompassing the brain and spinal cord without including somites (Tzouanacou et al., 2009). Additionally, the anterior region of the spinal cord must develop as a part of the anterior CNS without involving NMPs, as *Tbx6* ^−/−^ mutant embryos display NMP-dependent conversion of the paraxial mesoderm to extra neural tubes only at axial levels posterior to the seventh somite (Chapman and Papaioannou, 1998; Takemoto et al., 2011). Thus, anterior CNS domain specification appears to proceed similarly between chicken and mouse embryos, except that the mouse CNS precursors bypass the step of cell convergence toward the midline. Thus, mouse EpiSCs provide an excellent model for the early-stage specification of the anteroposterior regionality of the brain domains.

EpiSCs enter the neurogenetic process after removal of activin/Nodal signaling (Brons et al., 2007; Tesar et al., 2007; Iwafuchi-Doi et al., 2012). On the first day after activin removal, EpiSCs enter the transitory state between the epiblast and neurogenic states (Iwafuchi-Doi et al., 2012). We thus placed activin-free EpiSCs in medium containing EGF and FGF2, which may have resulted in the establishment of NSC lines representing an early stage of CNS development. We also manipulated the Wnt signal levels delivered to the NSC-generating EpiSCs using a Wnt antagonist and a Wnt agonist. We also manipulated the Wnt signal levels delivered to the NSC-generating EpiSCs using a Wnt antagonist and a Wnt agonist.

The activin-free, EGF and FGF2-supplemented culture conditions quickly changed the EpiSCs into neural cell populations in 1 week (Figures 2, 6), from which NSCs developed as cells adherent to gelatin-coated culture dishes (Figure 2). NSC lines with different morphological characteristics and culture requirements developed in 4 weeks (Figure 2B; Table 1). These were, in fact, NSCs, as they gave rise to neurons and glial cells under appropriate culture conditions (Figure 2C).

### Wnt signal-dependent anteroposterior regionality specification modeled in NSCs

Transcriptome analysis of the six NSC lines established under different Wnt signal inputs demonstrated that the characteristics of the NSC lines were ordered from anterior to posterior according to the Wnt signal input levels during the derivation of the cell lines. In the XN4 NSC line, where the Wnt signal input was the lowest with 10 µM XAV, *Foxg1*, characteristic of the anterior forebrain (Shimamura et al., 1997; Martynoga et al., 2002), was the prominent TF gene associated with low-level *Otx2* and *En2* expression (Figure 4A, Supplementary Figure S2). The XN1 line that developed under a lower concentration of XAV and hence a milder Wnt signal inhibition expressed *Otx2*, *En2*, *Hoxa5* (and other *Hoxa* genes with the anterior expression limit in the hindbrain), indicating that the regional specificity of the XN1 line covers the mid-hindbrain region.

KN3 and KN1 lines were established under culture conditions without a Wnt antagonist or agonist. This condition provided a unique situation in which cells responded to endogenous Wnt3 signaling. EpiSC-endogenous Wnt signaling was moderate and failed to activate *Bra* expression, whereas high *Bra* expression was induced by exogenous CHIR at 2 µM (Figure 6A). In addition, the EpiSC-endogenous Wnt signaling level likely fluctuates, as demonstrated for human ESCs (Moya et al., 2014), which resemble EpiSCs in many respects (Brons et al., 2007; Matsuda et al., 2017). KN3 cells expressed *En2* and cervical level-starting *Hoxa* genes more strongly than thoracic level genes and no *Hoxb* genes. In contrast, KN1 cells did not express *En2* but expressed high levels of cervical level *Hoxa5*, *6*, and *7* and anterior thoracic level-starting *Hoxa7* and *Hoxb* genes (Figure 4A). However, the KN1 cells did not express posterior thoracic level-starting *Hoxa9*. These observations indicated that the regional specificity of the KN3 cells covers the hindbrain to the cervical spinal cord, whereas that of the KN1 cells covers the cervical to the anterior thoracic level spinal cord. The differences in the regional coverages, although overlapping, are accounted for by the fluctuation of cell-endogenous Wnt signaling among EpiSCs.

The CN1 and CN4 NSC lines were established in the presence of 2 µM CHIR, which overrides endogenous Wnt signaling. The *Hox* gene expression patterns of the CN1 and CN4 lines were similar, presumably reflecting an equivalent level of exogenous Wnt signaling (Figure 4A). These NSC lines expressed thoracic level-characteristic *Hoxa9*, *Hoxa10*, and *Hoxc9* but did not express lumbar level-characteristic *Hoxc10* or *Hoxc11*. These observations indicate that the CN1 and CN4 NSC lines share regional specificities covering mainly the thoracic spinal cord.

During the derivation of CN4 or any other NSC lines, activation of the *Bra* gene was not detected (Figure 6A), indicating that NMPs are not involved in the derivation of these lines. However, the possibility of a transient activation *Bra* during the sampling intervals was not formally excluded. Presumably, the thoracic level spinal cord is the posterior limit of the CNS portion coverable by the NSCs derived from the *Otx2*-expressing (N2 enhancer-active) state of the epiblast; by increasing the CHIR concentration, no NSC outgrowth was observed. Spinal cord cells at the lumber and more posterior levels may develop fully from the NMPs. Presumably, in the range of thoracic levels, the origin of the spinal cord tissues gradually changes from the *Otx2*-expressing epiblast to NMPs. Concerning the chicken embryo case shown in Figure 1D, the posterior limit of the *Otx2*-expressing epiblast-derived spinal cord precursors is marked by the distribution of N2 enhancer-labeled cells.

### The early-stage characteristics and the anteroposterior regionalities in NSC lines

The anteroposterior regionalities of the above NSC lines already manifested after a week of culturing under the respective conditions (Figure 6Ac). These regionalities, once established, were maintained stably under culture conditions with different Wnt signal input levels, as shown in Figure 6B.

In contrast to the TF genes involved in the anteroposterior regional specification, those engaged in the dorsoventral specification in the neural tubes were expressed in a dysregulated fashion, presumably without any constraints (Figure 4), indicating that the NSCs were fixed at a stage earlier than when the neural plate begins to fold and the dorsoventral specification of the CNS cells starts. Interestingly, the expression of dorsal character TF genes, such as *Olig3* and *Pax3*, was generally very low. As Wnt signaling promotes dorsal side regional specification (Muroyama et al., 2002), the Wnt agonist CHIR would have promoted the expression of such genes if NSCs represented postneural plate developmental stages.

These observations were strongly in support of the following model: 1) the EpiSC-derived NSC lines produced by immediate exposure to the NSC-generating condition represent the neural precursor cell stage shortly after leaving the epiblast state; 2) at that stage, the gross anteroposterior regionalities are specified; and 3) the Wnt signal input levels in the cells are crucial to specifying the anteroposterior regionality. Thus, the early-stage anteroposterior regionality specification before neural plate development, as observed in chicken embryos (Figure 2A), was successfully modeled in mouse EpiSC-derived NSCs.

### Anteroposterior regionality range is associated with NSCs, a possible feature of the initial regional specification of neural primordia

The bulk transcriptome analysis of the NSCs summarized in Figure 4 indicated that individual cell lines were endowed with anteroposterior regional specificities reflecting the Wnt signal strength imposed during NSC derivation. However, the regional specificities had ranges. A critical question was whether these ranges reflect the composite of narrower specificity or reflect the character of individual cells.

The single-cell transcriptome analysis shown in Figures 7, 8 indicated that the NSC lines consisted of cells expressing TF genes of a given anteroposterior range. The study identified cells expressing one of the genes representing different regional characteristics within the regionality range and those expressing two or three such genes simultaneously. The statistical analysis indicated that the coexpression of multiple genes resulted from stochastic expression of individual genes without anteromedial or medioposterior regional correlations. A possible model to account for this observation is that individual cells have the potential to express all TF genes of the anteroposterior range characteristic of the cell line but express these genes transiently and stochastically, which may deserve further investigation.

Presumably, the characteristics of these NSC cells represent the initial phase of anteroposterior regionality specification in the neural primordium, although equivalent analyses have yet to be conducted using embryonic tissues. This study investigated six NSC lines that developed under discrete-level changes in Wnt signaling. In embryos, the Wnt signal levels will continuously change depending on the anteroposterior direction, and an array of neural primordia (possibly transient NSCs) bearing overlapping yet shifting regional specificities will be produced. These neural precursors may narrow their regional coverages in the cell population with slightly different regional specificities and during the course of neural plate formation.

The basic strategy in this study was to stabilize cells as NSC lines in a stage of neural development. We demonstrated that early-stage anteroposterior regionality is specified according to Wnt signal levels. In the mouse embryo CNS, various finer subdivisions are established by E11.5 (Bulfone et al., 1993; Shimamura et al., 1995; Shimamura et al., 1997; Gray et al., 2004; Berg et al., 2019; La Manno et al., 2021), which develop into functional CNS subdomains in adults (Thompson et al., 2014; Zeisel et al., 2018; Berg et al., 2019). These subdivisions will be generated under the intersections of various signaling pathways at different time points. The present approach may be widely useful in determining the elements of intersecting signaling and event chronology by changing the timing of exposure to the NSC-generating condition.

### Analogy with the anteroposterior regional specification of human PSC-derived neural crest cells

Anteroposterior regional specification reflecting differential Wnt signal input levels is not unique to neural plate precursors. Using human pluripotent stem cells, it has been shown that neural crest (NC) precursors are efficiently produced by 2 days of exposure to the Wnt antagonist CHIR, a condition for neural plate border cells (Thawani and Groves, 2020), and that NC cells develop after a 3-day incubation period (Leung et al., 2016; Gomez et al., 2019b; Heckland et al., 2019). Notably, lower-CHIR conditions enable the development of anterior characteristics, including *Otx2* expression in NC cells, while higher CHIR conditions elicit trunk-characteristic *Hox* gene-expressing NC development (Gomez et al., 2019a; Heckland et al., 2019). In the latter case, NC cells develop via NMPs coexpressing *Sox2* and *Bra* (Gomez et al., 2019a; Heckland et al., 2019), and combination with Fgf signaling enables the development of the posteriormost characters expressing *Hox13* paralogs (Heckland et al., 2019). The combination of high Wnt and FGF signaling is the condition needed to activate the *Sox2* N1 enhancer that regulates NMP cells (Takemoto et al., 2006; Takemoto et al., 2011).

The approach taken in our study was to stabilize the early stage of CNS precursors as NSC lines rather than promoting their progression into neural development. The time course of *Bra* and the *Hox* gene expression (Figure 6) indicated that the NSC lines likely did not pass through the NMP stage, possibly because these cells were incompatible with the EGF-FGF cosignaling condition. Overall, the anteroposterior regional specification processes appear to proceed analogously between the CNS and NC cells that develop in parallel in embryos, reflecting the Wnt signal input levels.

An important message derived from the study on human pluripotent stem cells by Leung et al. (2016) is that the Wnt signal has an additional role earlier than the anteroposterior regional specification of NC cells, i.e., to generate the NC cell lineage separable from the neural plate cell lineages in the presence of a sufficient level of the endogenous BMP signal; otherwise, human pluripotent stem cells develop into CNS cells (Leung et al., 2016). BMP signaling inhibits *Sox2* expression and neural plate development (e.g., Takemoto et al., 2006). In contrast, the entire population of mouse EpiSCs was found to develop into CNS cells two days after activin removal (Iwafuchi-Doi et al., 2012), indicating that EpiSC-derived cells exist under low-BMP conditions. This possible difference in the endogenous BMP level accounts at least partially for the lack of NC traits in the EpiSC-derived NSC lines investigated in this study.

## Conclusion

**FIGURE 9:**
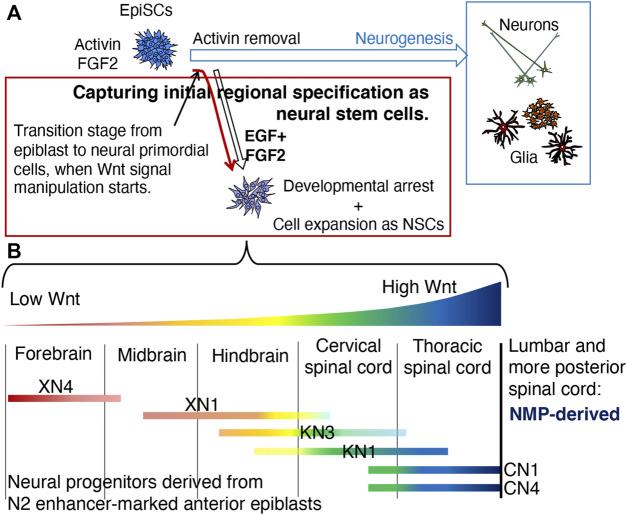
Summary of this study. **(A)** The strategy to capture the epiblast-to-neural transition stage and to assess the effect of differential Wnt signal levels on the anteroposterior regional specification of the neural primordia. Removing activin from the EpiSC culture medium initiates the neurodevelopmental process through a transition state on the first day of activin removal (Iwafuchi-Doi et al., 2012). Activin removal and placing EpiSCs in the EGF- and FGF2-continuing medium will generate NSC lines representing an early developmental stage of neural primordial cells. The manipulation of Wnt signal levels starting from the transition stage will result in the NSC lines representing the anteroposterior regional specification status of the early-stage stage neural primordia if the specification is under the control of Wnt signal strength. **(B)** Characterization of the NSC lines developed under different Wnt signal inputs. 1) The anteroposterior regional characteristics of the NSCs were arranged in the order of Wnt signal levels they were exposed to during NSC establishment; the lower, the more anterior, and the higher, the more posterior, confirming the participation of Wnt signal levels in the anteroposterior regional specification of the CNS. However, the initial regional specification represented by the NSC lines had an anteroposterior range, confirmed by the simultaneous expression of TF genes representing different anteroposterior levels. 2) The NSC lines that received a high level of Wnt signaling exhibited the characteristic gene expression profile of the thoracic SC but never those of lumbar and more posterior SC regions, indicating that the thoracic level is the posterior limit of regional characteristics attainable by the anterior epiblast-derived neural primordia. More posterior levels of SC likely develop from the NMP, which is derived from the posterior epiblast (Takemoto et al., 2006; Takemoto et al., 2011).

Figure 9 summarizes our strategy to capture the earliest stage of anteroposterior regional specification in the nascent neural primordium (Figure 9A) and its outcome (Figure 9B).

EpiSCs maintained in the activin- and FGF2-containing culture medium were freed from activin signaling to initiate neural development from EpiSCs via the transitory state that occurs a day after activin removal (Iwafuchi-Doi et al., 2012). The nascent neural primordial cells were placed in culture medium containing EGF and FGF2, a condition that expands early-stage neural primordial cells *in vitro* as NSCs. The activin-free cells were also exposed to different levels of Wnt signals to examine the possible involvement of Wnt signal levels in anteroposterior regional specification.

The six NSC lines established under different Wnt signal input levels were arranged in anterior-to-posterior order by CNS regionality, in concordance with the low to high Wnt signal input levels. However, the regionalities had anteroposterior ranges, indicated by the coexpression of TF genes associated with different anteroposterior levels. This feature may represent the nascent neural primordial state derived from the epiblast, although its validation using embryonic tissues is still needed. Thus, Wnt signal levels play determinative roles in the initial range specification of anteroposterior CNS regionalities; the regional specification in individual cells may be narrowed via cell‒cell interactions and additional molecular cues as neural development proceeds.

Even under a high Wnt signal level, represented by the CN1 and CN4 lines, the NSCs represented the thoracic level SCs but not the lumbar and more posterior levels. This result indicated that the thoracic SC is the posterior limit of the CNS tissues derived from the N2 enhancer-marked anterior epiblast in mouse embryos. SC of the lumbar and more posterior levels likely develops from NMPs derived from the posterior epiblast. The cellular mechanisms underlying the transition from anterior epiblast-derived SCs to posterior epiblast-derived SCs deserve future study.

## Materials and methods

### Chicken embryo electroporation and analysis of epiblast cell migration

St. 4 chicken embryos (Hamburger and Hamilton, 1951) isolated on a ring-shaped filter paper support were electroporated using the electrode setting described by Uchikawa et al. (2017) with 1 µg/µl N2-tkmCherry [a modified version of N2-tkmRFP1 (Uchikawa et al., 2017)] or a Supernova vector cocktail consisting of 1 µg/µL pK031 (TRE-CRE-pA), 0.5 µg/µL pK038 (CAG-LoxP-Stop-LoxP-GFP-IRES-tTA-pA) (Mizuno et al., 2014), and 0.4 µg/µL pTK-CAG-tTA (provided by Y. Takahashi) with four pulses of 8 V with 50 ms duration at 1 s intervals using CUY21SC (NEPAGENE, Ichikawa, Japan). The embryos were cultured on agar-albumen medium with the ventral side upward (Uchikawa et al., 2003; Yoshihi et al., 2022b). Time-lapse embryo images were recorded at 10-min intervals for 18 h under an AF6000 inverted microscope (Leica, Wetzlar, Germany). The trajectories of the Supernova-labeled cells were analyzed according to the method detailed by Yoshihi et al. (2022a).

### Mouse EpiSC and NSC cultures

EpiSC lines provided by Paul Tesar (Tesar et al., 2007; Iwafuchi-Doi et al., 2012) and Y62 line established from DBF1 epiblasts according to Sumi et al. (2013) were cultured under feeder-free conditions (Iwafuchi-Doi et al., 2012) and used to establish the NSC lines (Table 1). The basic culture medium was 50% DMEM (Sigma‒Aldrich, D6421)/50% Neurobasal Medium (Invitrogen, 21103-049), 0.004% BSA, with 2 mM L-glutamine, 0.5× N-2 (R&D, AR003) and 0.5 × B-27 (Invitrogen, 17504-044) supplements. The EpiSCs were cultured in basic culture medium with the addition of 20 ng/mL Activin A (PEPROTECH, 120-14), 10 ng/mL FGF2 (PEPROTECH, 100-18B), and 2 µM XAV939 and on a NunclonΔ surface coated with 10 µg/mL fibronectin (BD, 354008). NSCs were cultured in medium containing 10 ng/mL EGF (PEPROTECH, 100-15) and 10 ng/mL FGF2 in dishes coated with 0.2% gelatin (Sigma). When needed, 10 µM XAV939, 2 µM CHIR99021 (STEMGENT, 100-15), or 10 µM Y-27632 (Calbiochem, 146986-50-7) was added to the culture medium diluting the stock solutions prepared in dimethylsulfoxide (Table 1 and Supplementary Table S1). XAV939 was obtained from two different sources, Sigma‒Aldrich (X-3004) and Selleck (S1180). To transfer the cells, Accutase (Sigma or Nacalai) was used to dissociate cells except for the KN3 line, where the cell sheets were mechanically dissociated into cell clumps (Table 1). RNA was extracted from the cultures as described in Inamori et al. (2020). To differentiate the NSCs into neurons or glial cells, the dissociated cells were plated on uncoated dishes and cultured in basic culture medium for 3 days for neuronal development, or in medium containing 1% fetal bovine serum for 5 days for glial cell development. The cultures were fixed with 4% paraformaldehyde in PBS for 1 h and immunostained using the following combinations of primary and secondary antibodies: Tuj1 mouse monoclonal IgG (R&D MAB1195) and Alexa Fluor 488-donkey anti-mouse IgG (Abcam, ab150109); anti-O4 mouse monoclonal IgM (Sigma O7139) and Alexa Fluor 555-goat anti-mouse IgM (Invitrogen, A-21426); anti-GFAP rabbit polyclonal IgG (Santa Cruz SC-6170) and Alexa Fluor 568-donkey anti-goat IgG (Abcam, ab175704).

### Mouse E10.5 CNS tissues

FB, MB, HB, and CSC portions of the E10.5 CNS were isolated for comparison with the TF gene expression patterns of the NSC lines. The E10.5 stage was chosen because it is the developmental stage when the posterior-dominant Wnt expression profile is lost (Takemoto et al., 2016) and when the basic TF expression pattern in the CNS domains is established yet neurogenesis is minimal (Gary et al., 2004; Thompson et al., 2014; Andrews et al., 2019; Sagner and Briscoe, 2019). Two litters of E10.5 embryos of C57BL/6J mouse, with 5 and 6 embryos of the right stage, were dissected to isolate the embryonic portions containing FB, MB, HB, and the CSC (anterior to the forelimb bud). In PBS containing 10 μg/mL Dispase II (Wako) and 0.1% BSA, eyes, otic vesicles, and attached non-CNS tissues were removed. The separate CNS domains were pooled, and RNAs were extracted using TRIzol™ Plus RNA Purification Kit (ThermoFisher).

### Transcriptome analyses of cell populations

mRNA from biological replicates of the six NSC lines and four E10.5 CNS domains/portions were analyzed using SurePrint G3 Mouse GE Microarray 8 × 60K Ver. 2.0 (Agilent). The data were deposited in GEO with the accession number GSE237042. Principal component analysis of the data was performed using Subio software (Subio Inc., Amami, Japan). RT‒qPCR analysis of individual mRNA levels was performed as described in Inamori et al. (2020) using the primers described by Iwafuchi-Doi et al. (2012), except for Hoxb6 (Forward, TTC​CTA​TTT​CGT​GAA​CTC​CAC​CTT; Reverse, CCG​CAT​AGC​CAG​ACG​AGT​AGA) and Hoxa7 (Forward, ACG​CGC​TTT​TTA​GCA​AAT​ATA​CG; Reverse, GGG​TGC​AAA​GGA​GCA​AGA​AG) (Dickson et al., 2009).

### Single-cell transcriptome analysis

The NSC lines XN4, XN1, KN1, and CN4 in the growing phase were dissociated using Accutase, passed through a 20 µm cell strainer, suspended in 10x volumes of CellCover (Anacyte Laboratories) (Akiyama-Oda et al., 2022), and stored at 4°C for up to 2 weeks until the cells were processed for single-cell transcriptome library preparation. After washing in 50% DMEM/50% neurobasal medium with 0.004% BSA, the cells were loaded on a BD Rhapsody cartridge (BD Biosciences) to capture mRNAs of cells on isolated magnetic beads. Single-cell RNA sequencing libraries were prepared for each NSC line using a BD Rhapsody WTA Amplification kit (633801) according to the manufacturer-supplied protocol. The libraries were sequenced using Hi-SeqX (Illumina) for 150 b paired-end reads to gain Fastq data. The sequence data were processed using the BD Rhapsody WTA pipeline on the Seven Bridge Genomics platform (Tzani et al., 2021). R1 reads were used to gain transcript data in individual cells, and R2 reads were used to align them on the mouse mm10 (GRCm38) genome sequence. Then, spreadsheet data were produced for the captured transcript counts of each gene in each cell. The Seurat package in RStudio (v. 4.3.0) (Satija et al., 2015) was used to compare the four NSC lines using plots of uniform manifold approximation and projection (UMAP). After removing ribosomal protein and mitochondrial gene reads, the data for 4000 randomly selected cells from each NSC line library were normalized; then, the data were merged to compare the four cell lines. The transcriptome profiles in individual cells were analyzed using Modern CSV (https://www.moderncsv.com/) and Excel (Microsoft). The area-proportional Venn diagrams were drawn using eulerAPE (Micallef and Rodgers, 2014). The original and processed data were deposited to GEO with the accession number GSE239383.

## Data Availability

The datasets presented in this study can be found in online repositories. The names of the repository/repositories and accession number(s) can be found below: NCBI GEO under GSE237042 and GSE239383
