# Supplementary material for: Wnt signal-dependent antero-posterior specification of early-stage CNS primordia modeled in EpiSC-derived neural stem cells
Source: Front Cell Dev Biol. 2024 Feb 9;11:1260528. doi: 10.3389/fcell.2023.1260528 (PMC10884098; doi:10.3389/fcell.2023.1260528)
Supplement: Supplementary file 1 [file Table1.pdf]

Supplementary Table S1. Summary of neural stem cell derivation and differentiation

			Addition to the basal neural stem cell medium												
			10 μM XAV929					None				2 μM CHIR99021			
Experiment ID	Experimentor	Original EpiSC line (mouse line)	Derived NSC line	XAV source	Differentiation			Derived NSC line	Differentiation			Derived NSC line	Differentiation		
					N	O	A		N	O	A		N	O	A
0	CB	Tesar (129)	XN0	A	+	+	nd	KN0	+	+	nd	CN0	+	+	nd
1	KN	Tesar (129)	**XN1**	A	+	+	nd	**KN1**	+	+	+	**CN1**	+	+	nd
2	KN	Y62 (DBF1)	XN2	A	+	+	+	KN2	+	+	+	CN2	+	+	+
3	YW	Tesar (129)						**KN3**	+	+	+				
4	YW	Tesar (129)	**XN4**	B	+	+	+					**CN4**	+	+	+

XAV source: A, Sigma–Aldrich (X-3004), developing crystalline precipitates at 10 μM after overnight culturing (See **Supplementary Figure 1**).
B, Selleck (S1180) without crystalline precipitates at 10 μM.

Differentiation: N, Tuj1-positive neuronal cells; O, O4-positive oligodendrocytes; A, GFAP-positive astrocytes;
+, detected by immunofluorescence; nd, not determined.
