# Supplementary material for: Wnt signal-dependent antero-posterior specification of early-stage CNS primordia modeled in EpiSC-derived neural stem cells
Source: Front Cell Dev Biol. 2024 Feb 9;11:1260528. doi: 10.3389/fcell.2023.1260528 (PMC10884098; doi:10.3389/fcell.2023.1260528)
Supplement: Supplementary file 2 [file Image2.pdf]

Supplementary Figure S2

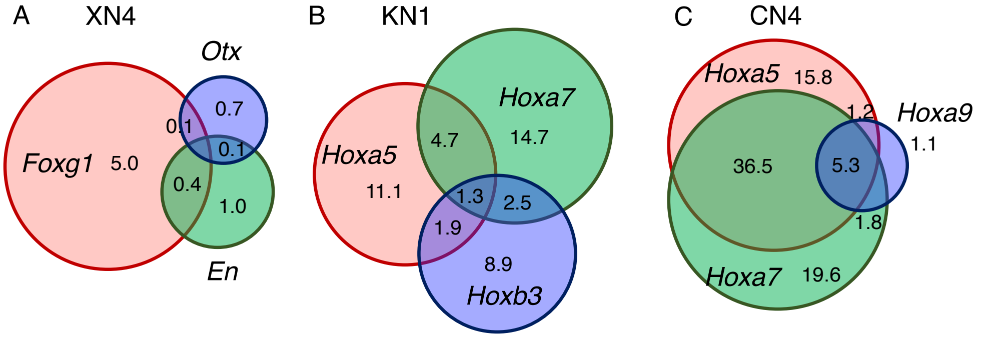

Supplementary Figure S2. **Statistics of cell fractions with transcript capture rates of single and multiple genes involved in the anteroposterior specification of the CNS primordia.** **A.** XN4 cell fractions expressing *Foxg1*, *Otx* genes, and *En* genes. As the transcript capture rates of *Otx2* and *En2* were very low, the transcript captures for *Otx2* or *Otx1* were combined as events for *Otx* capture. Similarly, *En2* or *En1* transcript captures were combined as *En* capturing to improve statistical significance. **B.** Analysis of *Hoxb3*, *Hox5a*, and *Hoxa7* gene transcripts in KN1 cells. **C.** Analysis of *Hoxa5*, *Hoxa7*, and *Hoxa9* gene transcripts in CN4 cells. Testing the model of the stochastic and independent expression of these TFs using these data, as shown in **Figure 8D**, resulted in the regression Line y =1.132x with R^2= 0.999.
