# Supplementary material for: Wnt signal-dependent antero-posterior specification of early-stage CNS primordia modeled in EpiSC-derived neural stem cells
Source: Front Cell Dev Biol. 2024 Feb 9;11:1260528. doi: 10.3389/fcell.2023.1260528 (PMC10884098; doi:10.3389/fcell.2023.1260528)
Supplement: Supplementary file 3 [file Image1.pdf]

Supplementary Figure S1

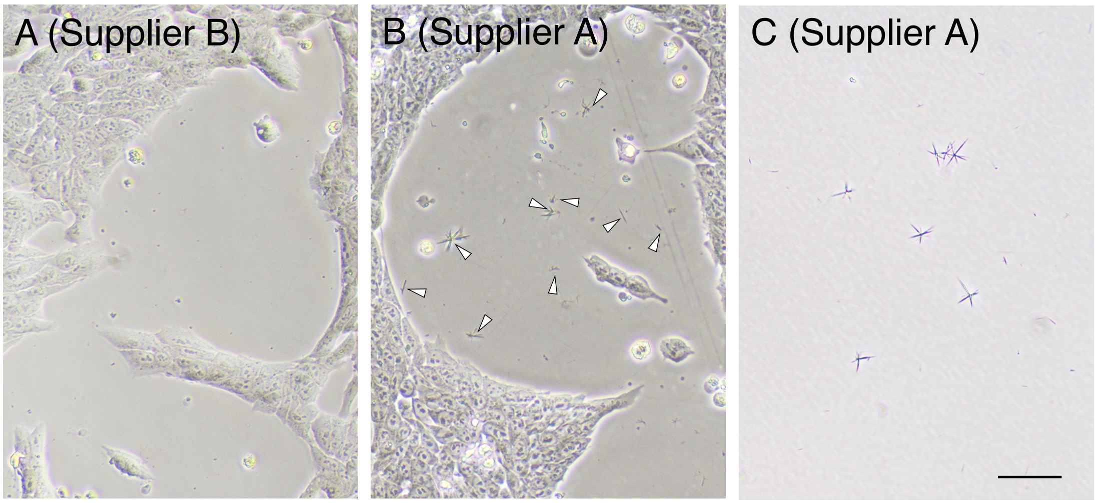

Supplementary Figure S1. Crystalline precipitate development using XAV obtained from supplier A added to the culture medium at 10 µM. A. Culture of EpiSCs with 10 µM XAV obtained from supplier B 24 h after plating the cells showed no development of XAV precipitates. **B.** Culture of sibling EpiSCs with 10 µM XAV obtained from supplier A. Many crystalline precipitates developed, as indicated by the arrowheads. A cell-free region of the culture surrounded by cells is shown to facilitate crystal detection. **C.** An aliquot of the same solution of XAV obtained from supplier A was diluted with the culture medium and incubated without cells, showing the crystalline precipitates in the form of radially arranged needles. The crystals became detectable microscopically after 2 h of incubation and developed further during the 24 h incubation period. The bar indicates 50 µm.

The difference in the 10 µM XAV obtained from suppliers A and B regarding the development of crystalline precipitates was highly reproducible. Our interpretation of this observation is as follows. At 10 µM, XAV in the culture medium at 37 °C is in an oversaturated yet metastable state. The impurities in XAV obtained from supplier A possibly act as nuclei for crystallizing the oversaturated portion of XAV. (According to the supplied data sheets, the purity is significantly higher for XAV obtained from supplier B). The effective concentration of XAV in the supernatant of the crystalline precipitates is equal to the saturation concentration of XAV in the culture medium.
